# ﻿Reconstitution of some tribes and genera of Lagriinae (Coleoptera, Tenebrionidae)

**DOI:** 10.3897/zookeys.1172.103149

**Published:** 2023-07-26

**Authors:** Rolf L. Aalbu, Kojun Kanda, Ottó Merkl, Michael A. Ivie, M. Andrew Johnston

**Affiliations:** 1 Department of Entomology, California Academy of Sciences, San Francisco, California, USA; 2 USDA Systematic Entomology Laboratory, c/o Smithsonian Institution, National Museum of Natural History, Washington, District of Columbia, USA; 3 Hungarian Natural History Museum, Department of Zoology, Budapest, Hungary; 4 Montana Entomology Collection, Montana State University, Bozeman, Montana, USA; 5 Biodiversity Knowledge Integration Center, Arizona State University, Tempe, Arizona, USA; † Deceased

**Keywords:** Darkling beetles, distribution, higher classification, identification keys, long-jointed beetles, nomenclature.

## Abstract

The tribes Goniaderini Lacordaire, 1859 and Lupropini Lesne, 1926 within the tenebrionid subfamily Lagriinae Latreille, 1825 have previously been shown to be non-monophyletic by molecular phylogenetic analyses. The tribes and constituent genera are here reviewed and redefined morphologically. As part of tribal redefinitions, we establish Prateini**New Tribe** with type genus *Prateus* LeConte, 1862. We reestablish the subtribe Phobeliina Ardoin, 1961 **Revised Status**, which is transferred from Goniaderini and placed as a subtribe of Lagriini Latreille, 1825 where it is comprised of *Phobelius* Blanchard, 1842, and *Rhosaces* Champion, 1889 (previously in Lagriini: Statirina Blanchard, 1845). The fossil tribe Archaeolupropini Nabozhenko, Perkovsky, & Nazarenko, 2023 is transferred from Lagriinae to Tetratomidae: Tetratominae Billberg, 1820. Keys to extant tribes and subtribes of Lagriinae and genera of Goniaderini, Lupropini, and Prateini are provided. Generic and species-level changes from this work are as follows:

Prateini is comprised of the following 15 genera: *Antennoluprops* Schawaller, 2007, *Ardoiniellus* Schawaller, 2013, *Bolitrium* Gebien, 1914, *Enicmosoma* Gebien, 1922, *Indenicmosoma* Ardoin, 1964, *Iscanus* Fauvel, 1904, *Kuschelus* Kaszab, 1982, *Lorelopsis* Champion, 1896, *Mesotretis* Bates, 1872, *Microcalcar* Pic, 1925, *Micropedinus* Lewis, 1894, *Paratenetus* Spinola, 1845, *Prateus*, *Terametus* Motschulsky, 1869, and *Tithassa* Pascoe, 1860. *Lorelus* Sharp, 1876 is **Returned to Synonymy** with *Prateus*, resulting in the following 49 **New Combinations**: *Prateusangulatus* (Doyen & Poinar, 1994), *P.angustulus* (Champion, 1913), *P.armatus* (Montrouzier, 1860), *P.biroi* (Kaszab, 1956), *P.blairi* (Kaszab, 1955), *P.brevicornis* (Champion, 1896), *P.breviusculus* (Champion, 1913), *P.caledonicus* (Kaszab, 1982), *P.carolinensis* (Blair, 1940), *P.chinensis* (Kaszab, 1940), *P.clarkei* (Kulzer, 1957), *P.crassicornis* (Broun, 1880), *P.crassepunctatus* (Kaszab, 1982), *P.cribricollis* (Kaszab, 1940), *P.curvipes* (Champion, 1913), *P.dybasi* (Kulzer, 1957), *P.fijianus* (Kaszab, 1982), *P.fumatus* (Lea, 1929), *P.glabriventris* (Kaszab, 1982), *P.greensladei* (Kaszab, 1982), *P.guadeloupensis* (Kaszab, 1940), *P.hirtus* (Kaszab, 1982), *P.ivoirensis* (Ardoin, 1969), *P.kanak* (Kaszab, 1986), *P.kaszabi* (Watt, 1992), *P.laticornis* (Watt, 1992), *P.latulus* (Broun, 1910), *P.longicornis* (Kaszab, 1982), *P.mareensis* (Kaszab, 1982), *P.marginalis* (Broun, 1910), *P.niger* (Kaszab, 1982), *P.norfolkianus* (Kaszab, 1982), *P.obtusus* (Watt, 1992), *P.ocularis* (Fauvel, 1904), *P.opacus* (Watt, 1992), *P.palauensis* (Kulzer, 1957), *P.politus* (Watt, 1992), *P.priscus* (Sharp, 1876), *P.prosternalis* (Kaszab, 1982), *P.pubescens* (Broun, 1880), *P.pubipennis* (Lea, 1929), *P.punctatus* (Watt, 1992), *P.quadricollis* (Broun, 1886), *P.queenslandicus* (Kaszab, 1986), *P.rugifrons* (Champion, 1913), *P.solomonis* (Kaszab, 1982), *P.tarsalis* (Broun, 1910), *P.unicornis* (Kaszab, 1982), and *P.watti* (Kaszab, 1982). *Microlyprops* Kaszab, 1939 is placed as a **New Synonym** of *Micropedinus* resulting in the following **New Combinations**: *Micropedinusceylonicus* (Kaszab, 1939) and *M.maderi* (Kaszab, 1940). *Lorelopsis***Revised Status** is revalidated as a genus and eight species formerly in *Lorelus* are transferred to it resulting in the following six **New Combinations**: *Lorelopsisbicolor* (Doyen, 1993), *L.glabrata* (Doyen, 1993), *L.exilis* (Champion, 1913), *L.foraminosa* (Doyen & Poinar, 1994), *L.minutulis* (Doyen & Poinar, 1994), *L.trapezidera* (Champion, 1913), and *L.wolcotti* (Doyen, 1993). *Lorelopsispilosa* Champion, 1896 becomes a **Restored Combination**.

In Goniaderini, *Aemymone* Bates, 1868 **Revised Status** and *Opatresthes* Gebien, 1928 **Revised Status**, which were recently considered as subgenera of *Goniadera* Perty, 1832, are restored as valid genera based on new character analysis resulting in the following **New Combinations**: *Aemymonehansfranzi* (Ferrer & Delatour, 2007), *A.simplex* (Fairmaire, 1889), *A.striatipennis* (Pic, 1934) and **Restored Combinations**: *Aemymonecariosa* (Bates, 1868), *A.crenata* Champion, 1893, and *A.semirufa* Pic, 1917. *Gamaxus* Bates, 1868 is **Returned to Synonymy** with *Phymatestes* Pascoe, 1866, and the type species *Gamaxushauxwelli* Bates, 1868 is placed as a **New Synonym** of *Phymatestesbrevicornis* (Lacordaire, 1859). The following seven genera are placed as **New Synonyms** of *Anaedus* Blanchard, 1842: *Microanaedus* Pic, 1923, *Pengaleganus* Pic, 1917, *Pseudanaedus* Gebien, 1921, *Pseudolyprops* Fairmaire, 1882, *Spinolyprops* Pic, 1917, *Spinadaenus* Pic, 1921, and *Sphingocorse* Gebien, 1921. Fourteen species described by Pic in *Aspisoma* Duponchel & Chevrolat, 1841 (not *Aspisoma* Laporte, 1833) are returned to Tenebrionidae as valid species of *Anaedus*. These synonymies necessitate the following 51 **New Combinations**: *Anaedusalbipes* (Gebien, 1921), *A.amboinensis* (Kaszab, 1964), *A.amplicollis* (Fairmaire, 1896), *A.anaedoides* (Gebien, 1921), *A.angulicollis* (Gebien, 1921), *A.angustatus* (Pic, 1921), *A.australiae* (Carter, 1930), *A.bartolozzii* (Ferrer, 2002), *A.beloni* Fairmaire, 1888), *A.biangulatus* (Gebien, 1921), *A.borneensis* (Pic, 1917), *A.carinicollis* (Gebien, 1921), *A.conradti* (Gebien, 1921), *A.cribricollis* (Schawaller, 2012), *A.gabonicus* (Pic, 1917), *A.himalayicus* (Kaszab, 1965), *A.inaequalis* (Pic, 1917), *A.jacobsoni* (Gebien, 1927), *A.lateralis* (Pic, 1917), *A.latus* (Pic, 1917), *A.longeplicatus* (Gebien, 1921) , *A.maculipennis* (Schawaller, 2011), *A.major* (Pic, 1917), *A.nepalicus* (Kaszab, 1975), *A.nigrita* (Gebien, 1927), *A.notatus* (Pic, 1923), *A.pakistanicus* (Schawaller, 1996), *A.pinguis* (Gebien, 1927), *A.punctatus* (Carter, 1914), *A.raffrayi* (Pic, 1917), *A.rufithorax* (Pic, 1917), *A.rufus* (Pic, 1917), *A.serrimargo* (Gebien, 1914), *A.sumatrensis* (Pic, 1917), *A.terminatus* (Gebien, 1921), *A.testaceicornis* (Pic, 1921), *A.testaceipes* (Pic, 1917), *A.thailandicus* (Schawaller, 2012), *A.trautneri* (Schawaller, 1994); and 13 restored combinations: *Anaedusboliviensis* (Pic, 1934), *A.claveri* (Pic, 1917), *A.diversicollis* (Pic, 1917), *A.elongatus* (Pic, 1934), *A.guyanensis* (Pic, 1917), *A.holtzi* (Pic, 1934), *A.inangulatus* (Pic, 1934), *A.inhumeralis* (Pic, 1917), *A.mendesensis* (Pic, 1917), *A.minutus* (Pic, 1917), *A.rufimembris* (Pic, 1932), *A.rufipennis* (Pic, 1917), *A.subelongatus* (Pic, 1932). The new synonymies with *Anaedus* necessitate the following six **New Replacement Names***Anaedusmaculipennis* (for *Spinolypropsmaculatus* Kulzer, 1954), *A.grimmi* (for *Aspisomaforticornis* Pic, 1917), *A.minimus* (for *Anaedusminutus* Pic, 1938), *A.merkli* (for *Anaedusdiversicollis* Pic, 1938), *A.ottomerkli* (for *Anaeduslateralis* Pic, 1923), *A.schawalleri* (for *Anaedusnepalicus* Schawaller, 1994).

*Capeluprops* Schawaller, 2011 is removed from Lupropini and provisionally placed in Laenini Seidlitz, 1895. *Plastica* Waterhouse, 1903 is transferred from Apocryphini Lacordaire, 1859 to Laenini. *Paralorelopsis* Marcuzzi, 1994 is removed from Lupropini and provisionally placed in Lagriinae incertae sedis. *Pseudesarcus* Champion, 1913 is transferred from Lagriinae incertae sedis to Diaperinae incertae sedis. *Falsotithassa* Pic, 1934 is transferred from Lupropini to Leiochrinini Lewis, 1894 (Diaperinae). *Mimocellus* Wasmann, 1904 is transferred from Lupropini to Tenebrionidae incertae sedis, and likely belongs in either Diaperinae or Stenochiinae.

***Dedication***

*During the preparation of this publication, one of us, our respected colleague and friend Ottó Merkl (1957–2021), passed away suddenly on his way to work. In his honor and in recognition of his help with this and other papers, we have named two species of* Anaedus *after him.*

## ﻿Introduction

The family Tenebrionidae Latreille, 1802 presently contains 2,307 valid genera placed in 12 subfamilies (Bouchard et al. 2021). The subfamily Lagriinae Latreille, 1825 contains 273 genera which represent ca. 12% of the entire family. Currently, we estimate the number of tenebrionid species to be more than 30,000 (Bouchard et al. 2021). Of these, more than 3,600 are placed in the subfamily Lagriinae.

The composition of lagriine tribes has been problematic for a long time. Many genera have been transferred to and from Lagriinae over the last 40 years. This has gradually led to some progress towards a better understanding of the subfamily and constituent tribes. The current subfamilial concept of Lagriinae is largely based upon the work of Watt (1974) who utilized both adult and larval morphology. Lagriinae can generally be diagnosed using the following characters: *adults* with only simple sensoria on antenna; labrum subquadrate to elongate; mandibular mola with few coarse ridges, not finely striate; procoxal cavities completely closed internally and externally; elytron with ten striae plus scutellary striole; hind wing without subcubital fleck; ovipositor coxite usually slender; gonostylus elongate, digitiform, situated apically; known *larvae* with 2-segmented antenna; body pubescent; antennal and mandibular bases separated by narrow strip of head capsule (Watt 1974; Tschinkel and Doyen 1980; Doyen and Tschinkel 1982; Doyen et al. 1990; Matthews and Bouchard 2008; Matthews et al. 2010).

Recent molecular studies have supported the monophyly of Lagriinae (Kergoat et al. 2014; Kanda et al. 2015; Aalbu et al. 2017), but also demonstrated that several tribes are not monophyletic. Based on the results of Kanda et al. (2015), Kanda (2016) transferred the South American genus *Chaetyllus* Pascoe, 1860 from Lupropini Lesne, 1926 to Laenini Seidlitz, 1895, the latter being previously considered a strictly Old World group. Kanda (2016) also described the genus *Grabulax* Kanda, 2016 as a second Neotropical genus of Laenini. Aalbu et al. (2017) used molecular sequence data to transfer *Eschatoporis* Blaisdell, 1906 out of Goniaderini Lacordaire, 1859 and re-established the monogeneric tribe Eschatoporini Blaisdell, 1906. Although these studies contributed towards establishing monophyletic tribes, the phylogenetic trees presented by them showed additional taxonomic issues throughout Lagriinae which have yet to be resolved.

**Figure 1.**
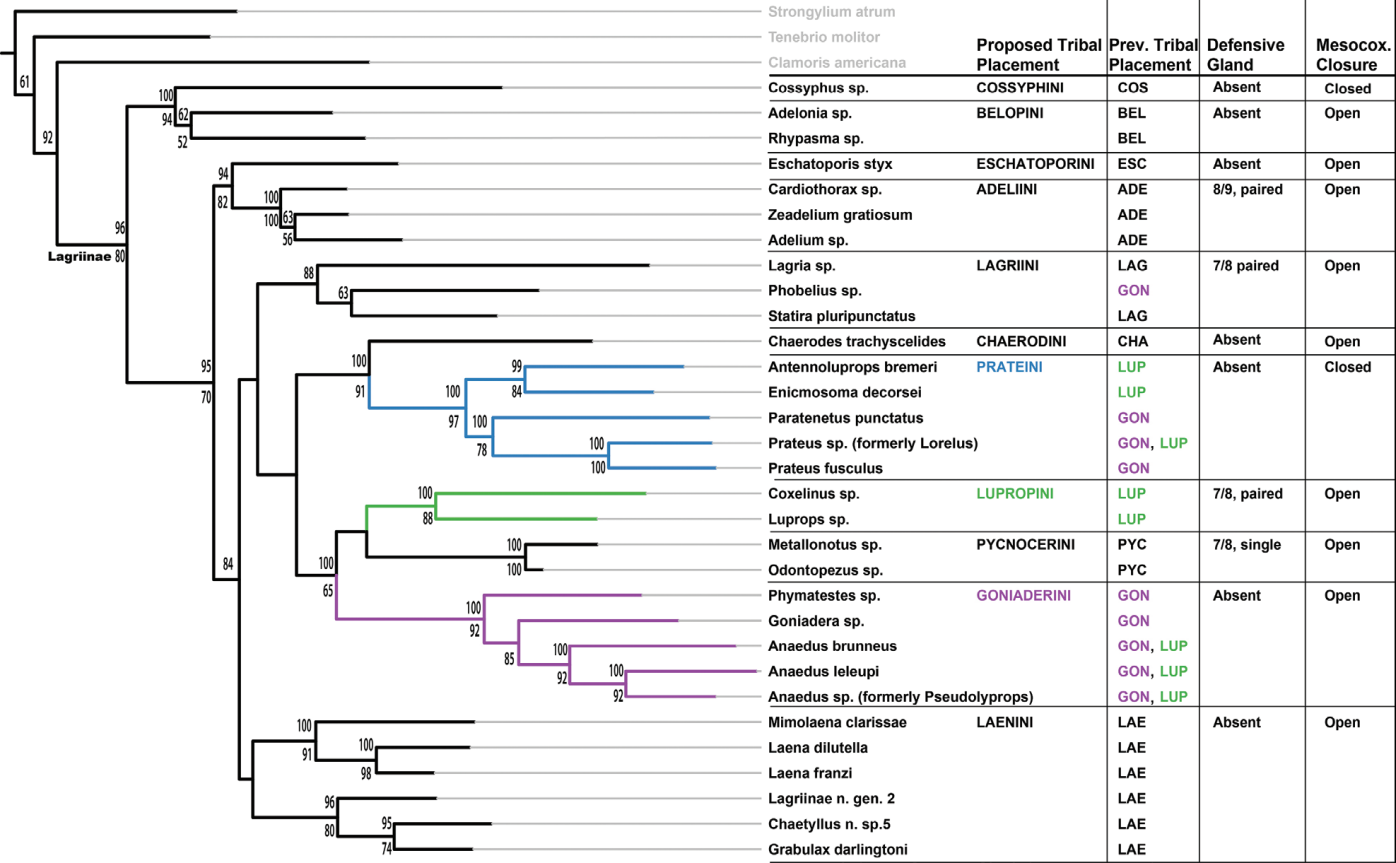
Phylogeny of Lagriinae from Aalbu et al. (2017) with revised generic names, tribal classification, and diagnostic morphological characters. Prev[ious]. Tribal Placement: COS = Cossyphini, BEL = Belopini, ADE = Adeliini, LAG= Lagriini, CHA = Chaerodini, LUP = Lupropini, GON = Goniaderini, PYC = Pycnocerini, LAE = Laenini. Defensive glands: location and number of abdominal defensive gland reservoirs: 8/9, paired = a pair of gland reservoirs with openings between abdominal sternites 8 and 9; 7/8, paired = a pair of gland reservoirs with openings between abdominal sternites 7 and 8; 7/8, single = single gland reservoir with opening between abdominal sternites VII and VIII. Mesocox[al]. Closure: Closed = lateral arms of meso- and metaventrites touching laterad of mesocoxa; Open = lateral arms not touching.

In the previously described studies, neither Goniaderini nor Lupropini were monophyletic, even after the taxonomic changes made in those papers (Fig. 1). Both are globally distributed tribes of predominantly litter-inhabiting and subcortical lagriines, which tend to be more diverse in tropical regions. These tribes are historically poorly defined and finding shared diagnosable characters among the taxa presently included in each has not been possible. In the molecular phylogenies presented in Kanda et al. (2015) and Aalbu et al. (2017), four genera that were classified in Goniaderini (*Lorelus* Sharp, 1876, *Phobelius* Blanchard, 1842, *Paratenetus* Spinola, 1845, and *Prateus* LeConte, 1862) are recovered outside of the clade containing *Goniadera* Perty, 1832 and related genera. *Phobelius* was recovered in Lagriini Latreille, 1825, and the remaining genera were recovered in a clade with two genera currently classified in Lupropini (*Enicmosoma* Gebien, 1922 and *Antennoluprops* Schawaller, 2007). A second clade of Lupropini was also recovered containing *Luprops* Hope, 1833 and *Coxelinus* Fairmaire, 1869. Although these issues were evident in the phylogenetic tree, the authors refrained from making taxonomic changes until a more detailed study of morphological characters could be conducted.

During independent work on the West Indian tenebrionid fauna, discrepancies between historic determinations of *Prateus* and *Lorelus*, by Theodore J. Spilman (1925–1996) and John T. Doyen (Doyen 1993; Doyen and Poinar 1994), led to the discovery of problems with the placement of *Prateus.* It became clear that the North American *Prateus* and West Indian *Lorelus* were congeneric and comparison with the New Zealand type species indicated this was correct. These two genera are currently placed in different tribes (Goniaderini and Lupropini respectively), clearly a problem. The resulting conclusion that *Prateus* was morphologically mischaracterized in its tribal placement caused a cascade of taxonomic and nomenclatural discoveries and an ever-widening set of issues, eventually with global implications. This fits with the problems of tribal definitions exposed by the molecular work, and this paper and two smaller taxonomic works (Ivie et al. 2021; Johnston et al. 2022) are the outcome.

In this study, we redefine Lupropini and Goniaderini, and establish a new tribe containing genera that were previously misclassified in the previous two. Keys to the genera in each of the three tribes and a key to the tribes of Lagriinae are provided. While examining material for this study, the need for new generic synonymies, and reversals of previous synonymies, were revealed. The tribal placements for several other genera are also fixed.

## ﻿Materials and methods

Specimens used in this study are deposited in the California Academy of Sciences, San Francisco, USA (**CASC**), United States National Museum of Natural History, Washington D.C., USA (**USNM**), Natural History Museum, London, UK (**NHMUK**), Hungarian Natural History Museum, Budapest, Hungary (**HNHM**), and Montana State University’s Michael A. Ivie collection (**MAIC**) and the West Indian Beetle Fauna Project, Montana State University (**WIBF**), as well as personal collections of Rolf Aalbu (**RLAC**), Kojun Kanda (**KKIC**), and Andrew Johnston (**MAJC**). Dissections and study of defensive glands were performed using protocols described by Tschinkel and Doyen (1980). In the following treatments of tribes, genera we could not examine are indicated with (*).

Specimens were examined with various stereomicroscopes. Photographs were made by use of the following systems: (1) Macropod Pro (Macroscopic Solutions), with a Canon EOS 5dsr camera body and 65mm lens. Images were stacked using Zerene Stacker v. 1.04. (2) A Nikon D5600 camera body mounted on a Stackshot rail system (Cognisys Inc.) equipped with a Laowa 60 mm or 25 mm macro lens. Images were stacked using Zerene Stacker v. 1.04. (3) A Zeiss Discovery.V20 Stereomicroscope with a Zeiss Axiocam 305 Color camera. Images were stacked using Zerene Stacker v. 1.04.

Morphological terminology follows Matthews et al. (2010) and Lawrence et al. (2011), though we prefer using the term “sternite” to homologously number the ventral sclerite of abdominal sections to “ventrite” which typically refers only to the externally visible abdominal sternites. We primarily employ external morphology along with internal characters of defensive glands and female ovipositors to diagnose the tribes below. Female genital tracts have shown great diagnostic utility across other tenebrionid groups but have not historically been used within Lagriinae (Watt 1974; Tschinkel and Doyen 1980; Doyen and Tschinkel 1982; Doyen et al. 1990; Matthews 1998). We only examined the internal tracts of a small fraction of taxa in this study but we were unable to discern any diagnostic trends or putative synapomorphies for the constituent groups. Bibliographic references are given for every species- and genus-group name treated in this study.

## ﻿Note on thoracic morphology

**Figures 2–5.**
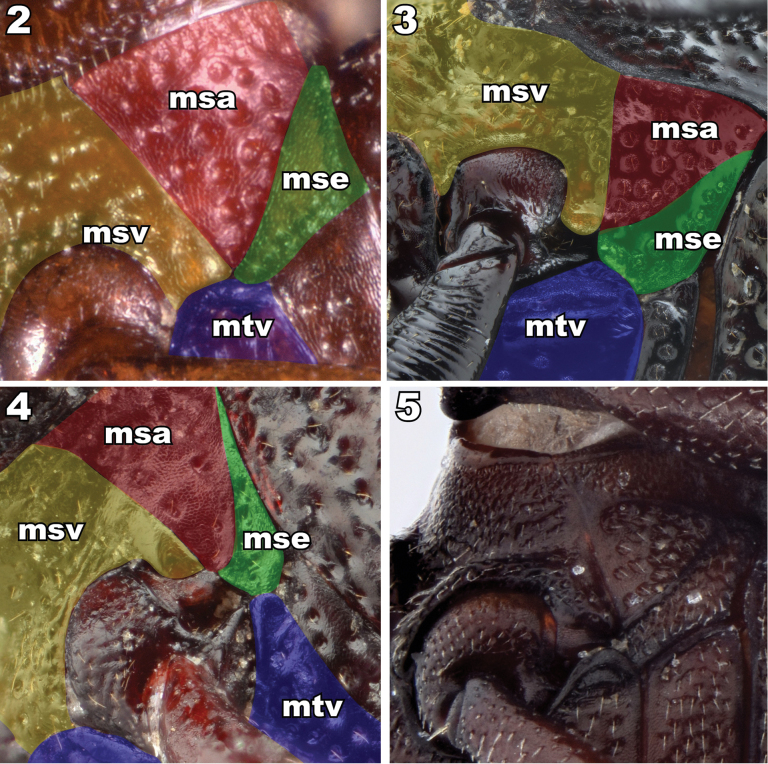
Mesocoxal closure in Lagriinae. **2** Closed state, *Prateusfusculus* LeConte, 1862 (Prateini) **3** Open state, *Phymatestes* sp. (Goniaderini) **4** Open state, *Capelupropslaenoides* Schawaller, 2011 (Laenini) **5** Closed state distorted during preservation, *Micropedinus* sp. (Prateini). Abbreviation: msv (yellow polygon) = mesoventrite, msa (red polygon) = mesanepisternum, mse (green polygon) = mesepisternum, mtv (blue polygon) = metaventrite.

One character system critical for the definition of the tribes involved herein requires explanation. The closure of the mesocoxal cavity is subject to misinterpretation (Figs 2–5). This character has four elements in this group (five in some non-focal taxa). They are the lateral arms of the meso- and metaventrites, the mesepimeron and the mesocoxa (the fifth being the mesanepisternum in some other taxa). The closed condition is usually defined by the lateral arms of the ventrites touching laterad the coxa (Fig. 2). The open condition is defined as the lateral arms not touching (Figs 3, 4), but this does not fully explain the true situation.

Under normal closed circumstances, the arms of the ventrites do clearly touch laterad the mesocoxa (Fig. 2). However, in the relatively soft-bodied taxa (for tenebrionids) involved here, the closure of the mesocoxal cavity may be subject to distortion, especially if the specimen has been dorso-ventrally compressed or the body distended in fluid preparation, resulting in the closure being “popped” open. The important part of this characteristic is not if the mesoventrite touches the metaventrite laterad the mesocoxa, but if the mesepimeron normally impinges into the space between them to reach the mesocoxae. In compressed and/or particularly soft specimens, the mesoventrite and metaventrite arms may be separated by a gap, but to be considered open, the mesepimeron requires a facing surface for contact with the ends of both arms, and the mesepimeron clearly reaches the mesocoxa. Some specimens, including some name-bearing type specimens, have this distorted condition, and this has led to historical misunderstanding and misplacement of taxa.

This “popped”-open condition is exemplified in Fig. 5 where there is a bead on the facing parts where the meso- and metaventrites normally touch. Though they are stretched apart, the mesepimeron does not extend between them to reach the mesocoxa. If you imagine the arms of the mesocoxae are moved back towards each other, the posterior face of the mesoventrite arm meets and conforms to the anterior face of the metaventrite arm, and the mesepimeron does not extend between them to reach the mesocoxa. Thus, the mesocoxal cavity is closed. Alternatively, when the meso- and metaventrite arms are clearly unable to meet because the tip of the mesepimeron touches the mesocoxa (Figs 3, 4), the mesocoxal cavity is truly considered open.

## ﻿Systematics

### ﻿ Prateini

Taxon classificationAnimaliaColeopteraTenebrionidae

tribe nov.

AB617461-5212-5F07-B22D-1190B9E8CBB3

https://zoobank.org/8D848712-F0ED-4A4C-982C-29C05298BCB8

Figs 6–14 , 15–18 , 19–20

#### Type genus.

*Prateus* LeConte, 1862.

#### Description.

Body length: 1.5–6.0 mm, stout to elongate, glabrous or setose. Most species are unicolored, fuscous to piceous, but a few species are patterned.

***Head***: Eyes round to ovoid, at most feebly notched anteriorly by epistomal canthus. Antennae usually reaching the middle of the pronotum, sometimes extending just past the base of the pronotum; antennomeres obconical to moniliform with last three to five forming a weak to strong club.

***Thorax***: Pronotum shape variable, usually quadrate to rectangular. Lateral margin complete, smooth to dentate. Procoxae clearly separated by prosternal process. Mesocoxal cavity laterally closed by meeting of lateral arms of meso- and metaventrite (Fig. 2). Elytra usually confusedly punctured, rarely with well-defined striae. Metathoracic wings usually well developed, but reduced or absent in some species. Legs slender, not fossorial. Penultimate tarsomeres lobed or cupuliform.

***Abdomen***: Intersegmental membranes visible between sternites V–VII, abdominal hinging tenebrionoid. Defensive glands absent. Ovipositor slender, with three to four clearly separated coxite lobes, terminal coxite digitate, gonostyli apical or subapical.

**Figures 6–14.**
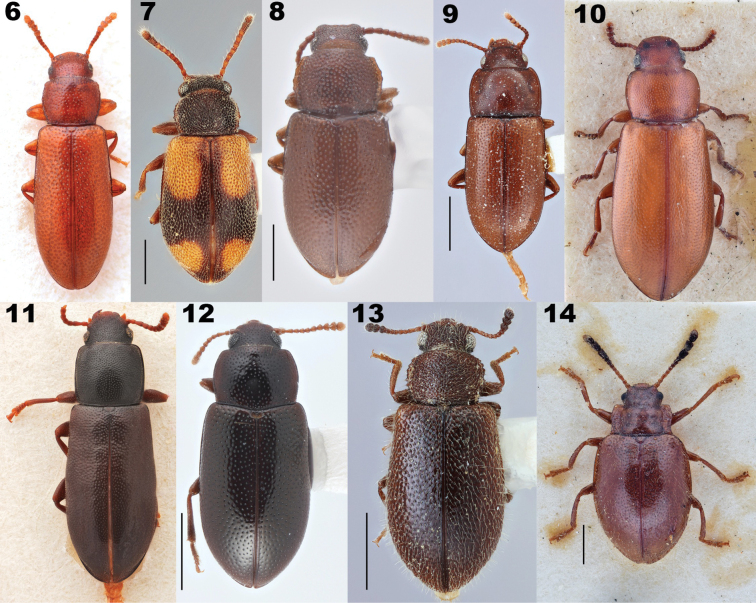
Dorsal habitus of representatives of Prateini genera. **6***Bolitriumchinensis* (Kaszab, 1940), holotype **7***Enicmosoma* sp. **8***Indenicmosomapunctator* Kaszab, 1979 **9***Iscanustrukensis* (Kulzer, 1957), paratype **10***Mesotretisferruginea* Bates, 1872, syntype **11***Microcalcarinstriatum* (Pic, 1925) **12***Micropedinus* sp. **13***Paratenetuspunctatus* Spinola, 1844 **14***Tithassacorynomelas* Pascoe, 1860. Scale bars: 0.5 mm (**7, 8**); 1 mm (**9, 12, 13**); images lacking scale bars were produced by Otto Merkl and sizes of specimens were not recorded before he passed.

#### Diagnosis.

Prateini is distinguished from Goniaderini and Lupropini by having the mesocoxal cavity closed (i.e., laterally closed by meeting of meso- and metaventrite) and absence of abdominal defensive glands.

In Lagriinae, this character combination is only shared with Cossyphini Latreille, 1802. These two tribes can easily be distinguished from each other by the general habitus; all species of Cossyphini have prominent pronotal and elytral flanges, and the pronotal flange covers the head. In Prateini, the pronotum never covers the head. Cossyphini also has medial hinging between abdominal sternites V–VII (i.e., tentyrioid hinging) and intersegmental membranes are not visible, while Prateini has lateral hinging between abdominal sternite V–VII (i.e., tenebrionoid hinging) and intersegmental membranes are visible.

#### Genera included.

*Antennoluprops* Schawaller, 2007a, Ardoiniellus* Schawaller, 2013, *Bolitrium* Gebien, 1914, *Enicmosoma* Gebien, 1922, *Indenicmosoma* Ardoin, 1964, *Iscanus* Fauvel, 1904, Kuschelus* Kaszab, 1982a, *Lorelopsis* Champion, 1896, *Mesotretis* Bates, 1872, *Microcalcar* Pic, 1925, *Micropedinus* Lewis, 1894, *Paratenetus* Spinola, 1845, *Prateus* LeConte, 1862, Terametus* Motschulsky, 1869 and *Tithassa* Pascoe, 1860.

**Figures 15–18.**
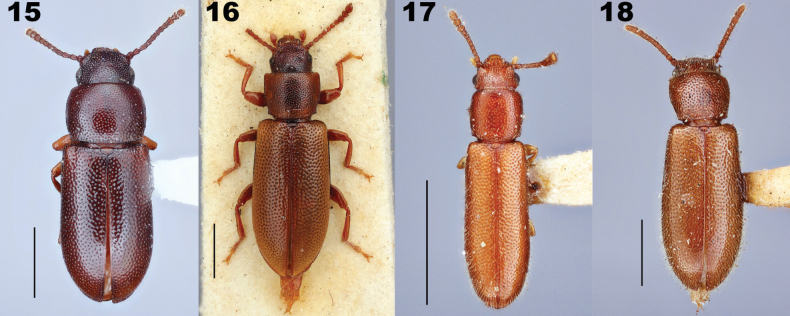
Dorsal habitus of species of *Prateus* LeConte, 1862 and *Lorelopsis* Champion, 1896. **15***Prateusfusculus* LeConte, 1862, type species of *Prateus***16***P.priscus* (Sharp, 1876), type species of *Lorelus* Sharp, 1876 **17***Lorelopsisexilis* (Champion, 1913) **18***L.trapeziderus* (Champion, 1913). Scale bars: 1 mm.

### ﻿Taxonomic changes among Prateini genera

#### ﻿Genus Prateus

Taxon classificationAnimaliaColeopteraTenebrionidae

LeConte, 1862

AFC0D311-07E6-5709-9E13-1251603A6D28

Prateus LeConte, 1862: 238. Type species: Prateusfusculus Leconte, 1862.
= Lorelus Sharp, 1876. Type species: Loreluspriscus Sharp, 1876. syn. rest. (original synonymy by Van Dyke 1953: 119).

##### Note.

The rarely collected *Prateusfusculus* of North America has not been critically studied since its description. Its placement in Goniaderini has led to the expectation that it has open meoscoxal cavities, but they are clearly closed (Fig. 3). This rediscovery was the impetus for this study. Blair (1940) first noted that *Prateus* and *Lorelus* “are very closely allied, if indeed really separable.” The synonymy was first proposed by Van Dyke (1953), who simultaneously described a second species, *P.dentatus* VanDyke, 1953, but was missed by Zoological Record, and remained unrecognized by all subsequent workers including recent catalogs (Bousquet et al. 2018; Bouchard et al. 2021). The synonymy was listed for the genus but no species-level combinations were proposed, which are now made explicitly below.

###### Species formerly assigned to *Lorelus*

*Prateusangulatus*† (Doyen & Poinar, 1994), comb. nov.

*Prateusangustulus* (Champion, 1913), comb. nov.

*Prateusarmatus* (Montrouzier, 1860) [*Trogosita*], comb. nov.

*Prateusbiroi* (Kaszab, 1956), comb. nov.

*Prateusblairi* (Kaszab, 1955), comb. nov.

*Prateusbrevicornis* (Champion, 1896), comb. nov.

*Prateusbreviusculus* (Champion, 1913), comb. nov.

*Prateuscaledonicus* (Kaszab, 1982b), comb. nov.

*Prateuscarolinensis* (Blair, 1940), comb. nov.

*Prateuschinensis* (Kaszab, 1940), comb. nov.

*Prateusclarkei* (Kulzer, 1957), comb. nov.

*Prateuscrassicornis* (Broun, 1880), comb. nov.

= *Lorelussternalis* Broun, 1910. Synonymy by Watt (1992).

*Prateuscrassepunctatus* (Kaszab, 1982b), comb. nov.

*Prateuscribricollis* (Kaszab, 1940), comb. nov.

*Prateuscurvipes* (Champion, 1913), comb. nov.

*Prateusdybasi* (Kulzer, 1957), comb. nov.

*Prateusfijianus* (Kaszab, 1982b), comb. nov.

*Prateusfumatus* (Lea, 1929) [*Mesotretis*], comb. nov.

*Prateusglabriventris* (Kaszab, 1982b), comb. nov.

*Prateusgreensladei* (Kaszab, 1982b), comb. nov.

*Prateusguadeloupensis* (Kaszab, 1940), comb. nov.

*Prateushirtus* (Kaszab, 1982b), comb. nov.

*Prateusivoirensis* (Ardoin, 1969), comb. nov.

*Prateuskanak* (Kaszab, 1986), comb. nov.

*Prateuskaszabi* (Watt, 1992), comb. nov.

*Prateuslaticornis* (Watt, 1992), comb. nov.

*Prateuslatulus* (Broun, 1910), comb. nov.

*Prateuslongicornis* (Kaszab, 1982b), comb. nov.

*Prateusmareensis* (Kaszab, 1982b), comb. nov.

*Prateusmarginalis* (Broun, 1910), comb. nov.

*Prateusniger* (Kaszab, 1982b), comb. nov.

*Prateusnorfolkianus* (Kaszab, 1982b), comb. nov.

*Prateusobtusus* (Watt, 1992), comb. nov.

*Prateusocularis* (Fauvel, 1904), comb. nov.

*Prateusopacus* (Watt, 1992), comb. nov.

*Prateuspalauensis* (Kulzer, 1957), comb. nov.

*Prateuspolitus* (Watt, 1992), comb. nov.

*Prateuspriscus* (Sharp, 1876), comb. nov.

*Prateusprosternalis* (Kaszab, 1982b), comb. nov.

*Prateuspubescens* (Broun, 1880), comb. nov.

*Prateuspubipennis* (Lea, 1929) [*Mesotretis*], comb. nov.

*Prateuspunctatus* (Watt, 1992), comb. nov.

*Prateusquadricollis* (Broun, 1886), comb. nov.

*Prateusqueenslandicus* (Kaszab, 1986), comb. nov.

*Prateusrugifrons* (Champion, 1913), comb. nov.

*Prateussolomonis* (Kaszab, 1982b), comb. nov.

*Prateustarsalis* (Broun, 1910), comb. nov.

= *Lorelusnigrescens* Broun, 1910. Synonymy by Watt (1992).

*Prateusunicornis* (Kaszab, 1982b), comb. nov.

*Prateuswatti* (Kaszab, 1982b), comb. nov.

#### ﻿Genus Micropedinus

Taxon classificationAnimaliaColeopteraTenebrionidae

Lewis, 1894

0392DDF4-A1C6-53B6-8F90-54E38C7341E8

Fig. 12

Micropedinus Lewis, 1894: 370. Type species: Micropedinusalgae Lewis, 1894.
= Notoprataeus Carter, 1924:37. Type species: Notoprataeuslitoralis Carter, 1924. Synonymy by Matthews and Lawrence (2005: 534).
= Microlyprops Kaszab, 1939: 108. Type species: Microlypropsceylonicus Kaszab, 1939. syn. nov.

##### Note.

This genus is known from littoral habitats in the Australasian, Indomalayan, and eastern Palearctic regions. The synonymy of *Microlyprops* was first suggested by Kaszab in his unpublished annotations within his physical copy of Gebien’s Catalog (Gebien 1941) complemented with handwritten remarks “[*Microlyprops*] *maderi* Kaszab […] = *Micropedinus* (Phaleriini), p. 497”. Moreover, he placed the *Microlyprops* specimens in the material of *Micropedinus* in the collection of the Hungarian Natural History Museum, Budapest. One of the co-authors, Otto Merkl, studied this material and confirmed the synonymy, but had not yet taken images before his passing. This synonymy results in the following species-group changes: *Micropedinusceylonicus* (Kaszab, 1939), comb. nov. and *Micropedinusmaderi* (Kaszab, 1940), comb. nov.

#### ﻿Genus Lorelopsis

Taxon classificationAnimaliaColeopteraTenebrionidae

Champion, 1896 stat. rev.

81801DC3-91BD-5ECB-A88C-AD662C4C7354

Figs 17–18 , 19–20

Lorelopsis Champion, 1896: 15. Type species: Lorelopsispilosa Champion, 1896.

##### Note.

Champion (1896) described this genus for a single species, *Lorelopsispilosa*, from St. Vincent in the Lesser Antilles, comparing it to *Lorelus*. He mentioned the lobe beneath the fourth tarsomere; pronotum narrower than elytra; erect, fine dorsal pubescence; and closed mesocoxal cavities as distinguishing generic characters. The only other species ever placed in *Lorelopsis* was when Wolcott (1936, 1951; see also Blackwelder 1945) mentioned an undescribed species from Yauco, Puerto Rico, determined by Chapin as belonging to this genus. Doyen (1993) described *Loreluswolcotti* Doyen, 1993 and listed Wolcott’s citation as a synonym but did not mention having actually seen the specimens cited by Wolcott. Doyen (1993) also stated that “*Lorelopsis* is probably not distinct from *Lorelus*.” Bouchard et al. (2021) record the two genera as synonyms, listing Doyen (1993) as a first synonymy ignoring the provisional nature of the statement.

We reestablish *Lorelopsis* as a valid genus in Prateini based upon several characters mentioned in the key and discussion below. Further, we move several species described in *Lorelus* by Champion (1913), Doyen (1993), and Doyen and Poinar (1994) to *Lorelopsis*. The new concept of this genus includes the species given in the checklist below, though a number of undescribed species are also known from the West Indies. Note that Champion (1896: 15) considered this genus to be masculine with his single described species ending in -*us* and this was followed by all subsequent workers through Bousquet et al. (2018). However, following ICZN Article 30.1.2, Bouchard et al. (2021) appropriately treated this genus as feminine and the species epithets are emended accordingly here.

*Lorelopsisbicolor* (Doyen, 1993), comb. nov.

*Lorelopsisglabrata* (Doyen, 1993), comb. nov.

*Lorelopsisexilis* (Champion, 1913), comb. nov.

*Lorelopsisforaminosa*† (Doyen & Poinar, 1994), comb. nov.

*Lorelopsisminutulis*† (Doyen & Poinar, 1994), comb. nov.

*Lorelopsispilosa* Champion, 1896, comb. rest.

*Lorelopsistrapezidera* (Champion, 1913), comb. nov.

*Lorelopsiswolcotti* (Doyen, 1993), comb. nov.

**Figures 19–20.**
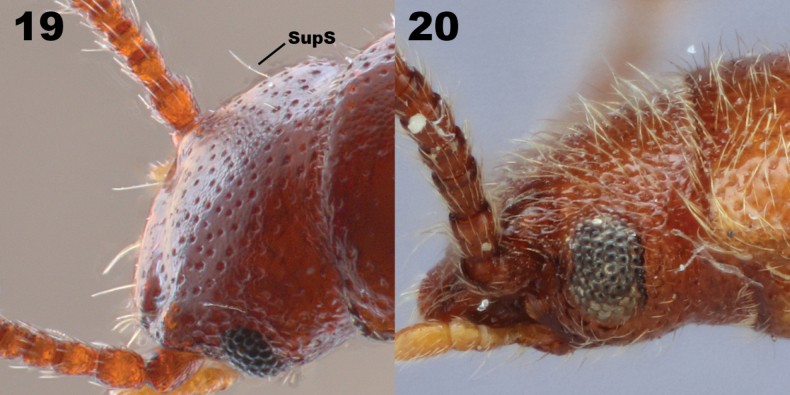
Head of two *Lorelopsis* species. **19***Lorelopsis* sp. with clearly discernible supraorbital setae (SupS) **20***L.trapeziderus* (Champion, 1913), a densely setose species in which the supraorbital setae are not discernible.

Besides having the characters of Prateini, *Lorelopsis* species are small, elongate, parallel-sided, and covered in fine, silky, erect to suberect setae. The pronotum is slightly to distinctly narrower than the base of the elytra and microspiculate on the lateral margin, each spicule with an associated projecting seta forming a fringing row of projecting setae. A distinct and newly observed character is a long, stout projecting seta on the dorsum of the head close to the hind edge of the eye. This seta is clearly visible in species with relatively sparse and short setae on the head (Fig. 19) but becomes less distinct when more dense and longer setae are present, blending with others (Fig. 20). Since not all species assigned here have been examined (specifically several of the Champion species from the mainland), and since this character has not previously been mentioned, it is possible that it does not occur in all the mainland species, but it is there in the species we have seen. Some, but not all, species have the fourth tarsomere lobed beneath for a variable length. Champion used this as a primary character when he described the genus, but it has proved to be a species-level character.

#### ﻿Key to the genera of Prateini

**Table:** 

1	Antenna with 10 antennomeres	**2**
–	Antenna with 11 antennomeres	**4**
2	Eye oval in lateral view, never prominent, narrowed by gena; pronotum with lateral margins of disc dorso-ventrally flattened; antennae highly and differently modified between sexes [Madagascar] (see Schawaller 2007a)	***Antennoluprops***
–	Eye round, prominent, sometimes conical; pronotal disc convex to lateral margins, not flattened at sides; antennae unmodified in both sexes	**3**
3	Body surface pubescent [Tropical Africa, Madagascar] (Fig. 7)	***Enicmosoma***
–	Body surface glabrous [Southeast Asia, India] (Fig. 8; also see Schawaller and Bigalk 2020; Ardoin 1964)	***Indenicmosoma***
4	Body oval, posterior margin of pronotum extended medially, closely appressed to elytra; elytral epipleuron very wide, nearly as wide as width of metacoxa [South Africa] (see Schawaller 2007b; Motschulsky 1869)	***Terametus***
–	Body not oval, posterior margin of thorax not extended medially; elytral epipleuron much narrower than width of metacoxa	**5**
5	Front edge of clypeus produced and very shallowly emarginate at middle; tarsi short and broad, all preapical tarsomeres transverse; antenna short, not reaching middle of pronotum. Length: 3–4 mm. [Australia on beaches] (Fig. 10; also see Matthews and Bouchard 2008; Bates 1872)	***Mesotretis***
–	Front edge of clypeus produced or not, but never emarginate; tarsi narrower, at least some preapical tarsomeres longer than wide; antenna reaching middle of pronotum	**6**
6	Labial palp with terminal palpomere larger than subterminal; antennae from 7^th^ antennomere to end gradually widened; body length: 1.2–5.4 mm [SE Asia, Australia, Pacific on beaches] (Fig. 12; also see Lewis 1894)	***Micropedinus***
–	Labial palp with subterminal palpomere enlarged, terminal palpomere small and parallel-sided; antenna more abruptly broadened from antennomere 8 or 9, last 3–4 ca. equal in width	**7**
7	Scutellum not visible; species very small (1.8 mm) [New Caledonia] (see Kaszab 1982b)	***Kuschelus***
–	Scutellum usually visible (except one species of *Prateus*); species larger (> 2 mm)	**8**
8	Body surface fully pubescent; pronotum laterally setose	**9**
–	Body glabrous or partially pubescent; pronotum laterally asetose	**11**
9	Eye very large, distance between eye and anterior edge of pronotum dorsally less than length of eye [North and South America] (Fig. 13; see also Bousquet and Bouchard 2014)	***Paratenetus***
–	Eye much smaller, distance between eye and anterior edge of pronotum dorsally equal or greater than length of eye	**10**
10	Body very elongate, parallel sided; head with a long stout seta dorsally near posterior edge of eye (obscured by other long setae when head is more densely setose); distance between eye and anterior edge of pronotum dorsally ca. equal to length of eye; pronotum laterally microspiculate; elytra not globose. [Tropical America] (Figs 17, 18; also see Champion 1896)	***Lorelopsis***
–	Body rounded, wide; head without stout seta above eye; distance between eye and anterior edge of pronotum dorsally usually much greater than length of eye; pronotum distinctly constricted posteriorly; elytra globose [tropical America] (Fig. 14)	***Tithassa***
11	Pronotal base slightly narrower than elytral base, humeri rounded; body setose or not	**12**
–	Pronotal base not or barely narrower and elytral base, humeri variable, often sub-angulate; body always glabrous	**13**
12	Antennal club with 3 antennomeres [widespread, mainly tropical Asia, Pacific, and Americas] (Figs 15, 16)	***Prateus***
–	Antennal club with 4 antennomeres [Southeast Asia] (Fig. 6; also see Bremer 1995,^1998^)	***Bolitrium***
13	Humeri angulate, not rounded; eyes small; basal membrane of labrum covered by broadened edge of clypeus. [Pacific Islands] (Fig. 9; also see Matthews and Bouchard 2008; Fauvel 1904)	***Iscanus***
–	Humeri clearly rounded, not angulate; eyes larger; basal membrane of labrum visible or not	**14**
14	Head with transverse impression between eyes; small species (1.8–2.5 mm) [South Africa] (see Schawaller 2013)	***Ardoiniellus***
–	Head without transverse impression between eyes; larger species (4–5.5 mm) [Madagascar] (Fig. 11)	***Microcalcar***

#### ﻿Tribe Goniaderini

Taxon classificationAnimaliaColeopteraTenebrionidae

Lacordaire, 1859

1E96E781-4769-59AF-910F-266B1F67271B

Figs 21–28 , 29 , 30–41

##### Type genus.

*Goniadera* Perty, 1832.

##### Description.

Body length: 3–19 mm; stout to elongate, dorsoventrally flattened to having elytra strongly inflated, glabrous or setose. Most species are unicolored, some are bicolored (e.g., pronotum and elytra with different coloration) or have patterned elytra.

***Head***: Eyes reniform, anteriorly notched by canthus, rarely completely divided. Antennae moderately long, usually reaching past base of pronotum; antennomeres obconical to filiform.

***Thorax***: Pronotum shape variable, usually cordate, constricted at base, sometimes quadrate to rectangular. Lateral margins complete. Procoxae clearly separated by prosternal process. Mesocoxal cavity laterally closed, at least partially, by mesepimeron. Elytra striate or not. Metathoracic wings well developed (in all species examined by us). Legs slender, not fossorial, penultimate tarsomeres lobed or cupuliform.

***Abdomen***: Intersegmental membranes visible between sternites V–VII, abdominal hinging tenebrionoid. Defensive glands absent. Ovipositor either stout with four distinct gonocoxites and terminal gonocoxite digitate or greatly reduced with gonocoxites fused (e.g., *Anaeduspunctatus* (Carter, 1914) see Matthews and Bouchard 2008).

##### Diagnosis.

Goniaderini can be distinguished from Lupropini and Prateini by having the mesocoxal cavities laterally open (i.e., laterally, at least partially closed by mesepimeron) and abdominal defensive glands absent.

In Lagriinae, this combination of characters is shared with Belopini Reitter, 1917, Chaerodini Doyen, Matthews & Lawrence, 1990, Eschatoporini, and Laenini (Fig. 1). Goniaderini can be distinguished from these tribes as follows.

In Belopini, abdominal hinging between sternites V–VII is medial (tentyrioid hinging), and no intersegmental membrane is visible between the sternites; the aedeagus is oriented so the tegmen is ventral, as in the majority of Pimeliinae; penultimate tarsomeres are not lobed or cupuliform. Goniaderini has lateral abdominal hinging between sternites V–VII (tenebrionoid hinging), and the intersegmental membranes between these segments are visible; aedeagus is oriented so the tegmen is dorsal; penultimate tarsomere is either lobed or cupuliform.

Chaerodini contains just two genera found on sandy shores in Australia and New Zealand. They exhibit features typical of psammophiles, including having a globose body, fossorial protibiae, and shortened antennae. Chaerodini also has an antennal club composed of five antennomeres and very reduced ovipositors that lack apical gonostyli. Goniaderini is not globose, at most only the elytra are inflated; protibiae are not fossorial; and antennae extend past the anterior margin of the pronotum and are not clubbed. The ovipositor is shortened and reduced in some groups (e.g., *Anaedus* Blanchard, 1842), but gonostyli are always present.

**Figures 21–28.**
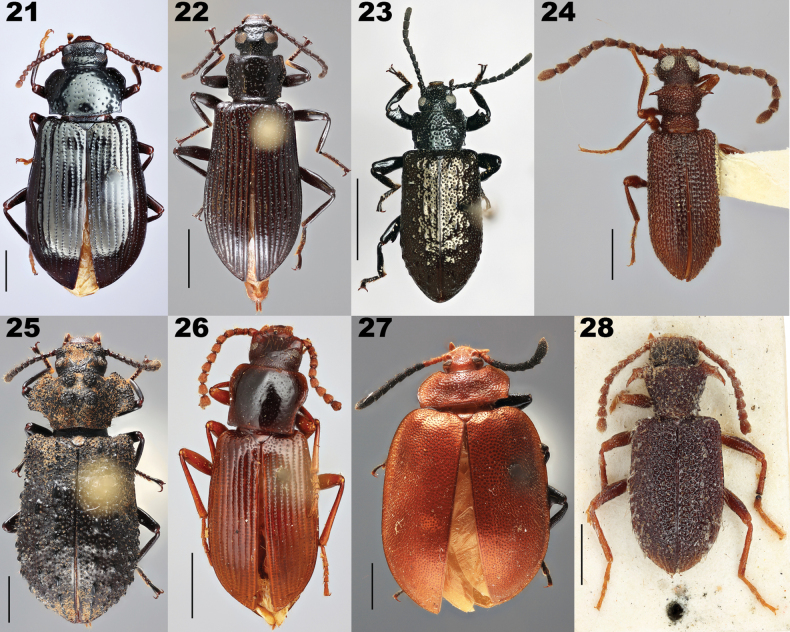
Dorsal habitus of representatives of Goniaderini genera. **21**) *Aemymonecariosa*Fairmaire 1873**22***Goniaderarepanda* (Fabricius, 1801) **23***Phymatestesspathifer* Gebien, 1928 **24***Spinolagriella* sp. **25***Opatresthesquadrinodosus* Gebien, 1928 **26***Xanthiclescaraboides* Champion, 1886 **27***Lyprochelyda* sp. **28***Ancylopomapunctigera* Pascoe, 1871, holotype. Scale bars: 1 mm (**22, 26**); 2 mm (**19, 23–25**); 5 mm (**20, 21**).

Eschatoporini contains just one genus with two species restricted to Northern California. These species inhabit caves with natural water and are sometimes found at entrances to underground springs. The eyes are completely absent. Goniaderini possesses well-developed reniform eyes. Although Eschatoporini and Goniaderini both lack sternal defensive glands, the former possesses a pair of cuticular sac-like reservoirs between tergites VII and VIII. This character seems to be unique within Tenebrionidae, and their function is unknown (Aalbu et al. 2017).

Most Laenini has small, rounded eyes that are not anteriorly notched by the epistomal canthus; body shape elongate, semi cylindrical but with strong constriction between thorax and abdomen making thorax rounded and abdomen elongate rounded; all species are apterous. Goniaderini has reniform eyes that are anteriorly notched by the epistomal canthus and although the body shape is highly variable, all examined species are winged.

##### Genera included.

Acropachia* Mäklin, 1875, *Aemymone* Bates, 1868, *Anaedus* Blanchard, 1842, *Ancylopoma* Pascoe, 1871, *Goniadera* Perty, 1832, *Lyprochelyda* Fairmaire, 1899, Microgoniadera* Pic, 1917a, *Myrmecopeltoides* Kaszab, 1973, *Opatresthes* Gebien, 1928, *Phymatestes* Pascoe, 1866, *Spinolagriella* Pic, 1955, and *Xanthicles* Champion, 1886.

###### ﻿Taxonomic changes among Goniaderini genera

Ferrer and Delatour (2007) revised the genera *Goniadera* and *Microgoniadera*, and placed both *Aemymone* and *Opatresthes* as subgenera of *Goniadera* mainly based on external surface characters. The characters listed in the former work to diagnose the tribe Goniaderini included mostly generalized lagriine or other variable characters. Their tribal concept also included *Eschatoporis* (Eschatoporini, see Aalbu et al. 2017). No other genus was mentioned other than *Microgoniadera*, which was separated in their key as a distinct species based only on size. They did not consider *Anaedus* to belong to Goniaderini but rather to Lupropini.

*Anaedus* clearly belongs morphologically within Goniaderini, which is consistent with molecular analyses (Aalbu et al. 2017). In fact, *Aemymone* (Fig. 19) is likely more closely related to *Anaedus* due to both possessing very elongate basal hind tarsomeres (not mentioned by Ferrer and Delatour 2007) as well as a lack of tubercles. Size is not reliable as certain species of *Anaedus*, like *An.robusticollis* (Pic, 1921), are larger than most *Aemymone. Aemymone* differs from *Anaedus* by (1) having clearly defined, punctate elytral striae, (2) lacking posterior pointing denticles on the lateral margin of elytra near the base, and (3) by having a slight metallic sheen in some species.

Ferrer and Delatour (2007) separated *Goniadera* and *Opatresthes*, as subgenera in their work, based upon the presence of setae (we find that both genera have setae), color of the integument (we find this character unreliable), and the sides of pronotum (we find this character reliable, although not adequately described in their key). Both *Goniadera* and *Opatresthes*, unlike *Aemymone*, have the basal tarsomere of the hind tarsi equal or subequal to the terminal tarsomere. These two genera can be further separated from each other by (1), the strongly explanate anterior two-thirds of the pronotum in *Opatresthes* (only at most slightly explanate sides of the pronotum in *Goniadera*), (2) the lateral aspect of both the pronotum and elytra being strongly dentate/tuberculate in *Opatresthes* (lateral aspect at most with a few dentitions on the pronotum in *Goniadera*), (3) the metaventrite is equal to or shorter than the first visible abdominal ventrite in *Opatresthes* (metaventrite longer than length of first abdominal ventrite in *Goniadera*), and (4) general shape, *Goniadera* being narrower and more elongate than *Opatresthes*.

The reinstatement of *Aemymone* and *Opatresthes* is summarized in the following checklists. Note that many authorship and year attributions of Ferrer and Delatour (2007) were incorrect.

#### Genus Aemymone

Taxon classificationAnimaliaColeopteraTenebrionidae

Bates, 1868 stat. rev.

14C9E077-1B5C-5D3C-A6F9-355F7401F975

Fig. 21

Aemymone Bates, 1868: 314. Type species: Goniaderacariosa Bates, 1868.

##### List of Aemymone species.

*Aemymonecariosa* (Bates, 1868) [*Goniadera*], comb. rest. Note: Bates (1868) described *Goniaderiacariosa* Bates, 1868, for an unavailable Dejean species of the same name, and later in the same paper designated this species as the type species for *Aemymone*. Gebien (1941) lists the type species simply as “*cariosa*”, but the only species with that epithet he included within the genus was one described by Fairmaire (1873). This was followed by Blackwelder (1945) and Ferrer and Delatour (2007) who also neglected to refer to Bates’ species. Bousquet et al. (2018) and Bouchard et al. (2021) recognized the proper Bates type species. See *Aemymonestriatipennis* below.

*Aemymonecrenata* Champion, 1893, comb. rest.

= *Goniaderachampioni* Ferrer & Delatour, 2007. Replacement name due to secondary homonym. Note: When Ferrer and Delatour (2007) included *Aemymone* as a subgenus of *Goniadera*, this resulted in *Goniaderacrenata* (Champion, 1893) [*Aemymone*] becoming a secondary homonym of *Goniaderacrenata* Perty, 1832. *Goniaderachampioni* Ferrer & Delatour, 2007 was proposed as a replacement name for *G.crenata* (Champion, 1893).

*Aemymonehansfranzi* (Ferrer & Delatour, 2007) [*Goniadera*], comb. nov.

*Aemymonesemirufa* Pic, 1917a, comb. rest.

*Aemymonesimplex* (Fairmaire, 1889) [*Goniadera*], comb. nov.

= *Aemymonebordoni* Marcuzzi, 1994. Synonymy by Ferrer and Delatour (2007).

*Aemymonestriatipennis* (Pic, 1934) [*Anaedus*], comb. nov. Synonymy with *A.cariosa* Fairmaire, 1873 by Ferrer and Delatour (2007).

= *Goniaderacariosa* Fairmaire, 1873. Junior primary homonym (in *Goniadera*) and secondary homonym (in *Aemymone*) of *Goniaderacariosa* Bates, 1868.

= *Aemymonesilvanae* Marcuzzi, 1994. Synonymy by Ferrer and Delatour (2007).

##### Note.

As noted above, *Goniaderacariosa* Fairmaire, 1873 is a primary homonym of *Goniaderiacariosa* Bates, 1868, and now that both species are included in *Aemymone*, it is also a secondary homonym. Although both species may have been described to accommodate an unavailable Dejean species by the same name (Bates 1968; Ferrer and Delatour 2007), the original descriptions suggest that each author formulated their description based on different specimens. To deal with the homonymy, *Aemymonestriatipennis* (Pic, 1934), which was synonymized by Ferrer and Delatour (2007) with *Aemymonecariosa* (Fairmaire, 1873) is considered the valid name. Type specimens of both species must be examined before a decision can be made about whether Fairmaire’s *A.cariosa* is a subjective synonym of Bates’.

#### ﻿Genus Opatresthes

Taxon classificationAnimaliaColeopteraTenebrionidae

Gebien, 1928 stat. rev.

6A970DCD-0061-5723-AB58-FDFF92646381

Fig. 25

Opatresthes Gebien, 1928: 192. Type species: Opatresthesbinodosa Gebien, 1928.

##### List of Opatresthes species.

*Opatresthesbinodosa* Gebien, 1928, comb. rest.

*Opatresthesquadrinodosa* Gebien, 1928, comb. rest.

*Opatresthesmaesi* (Ferrer & Delatour, 2007) [*Goniadera*], comb. nov.

*Opatresthestuberculifera* (Fairmaire, 1889) [*Goniadera*], comb. nov.

#### ﻿Genus Phymatestes

Taxon classificationAnimaliaColeopteraTenebrionidae

Pascoe, 1866

8910DDAD-787A-5B9D-A091-95BEE7E48EC5

Figs 23 , 29

Phymatestes Pascoe, 1866: 142. Type species: Lagriatuberculata Fabricius, 1787.
= Gamaxus Bates, 1868: 315. Type species: Gamaxushauxwellii Bates, 1868. syn. rest. (original synonymy by Gebien 1928: 191).

##### Note.

**Figure 29.**
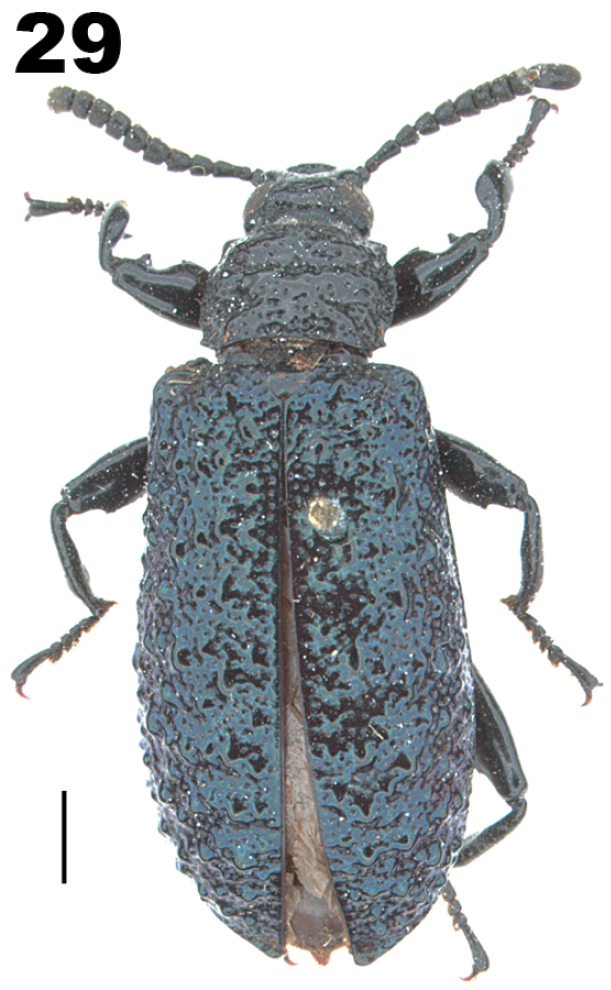
Dorsal habitus of holotype of *Gamaxushauxwellii* Bates, 1868 [=*Phymatestesbrevicornis* (Lacordaire, 1859)]. Scale bar: 2.5 mm.

Bates (1868: 315) distinguished *Gamaxus* (Fig. 29) from *Phymatestes* (Fig. 23) by havinG “shorter antennae with apical antennomeres strongly transverse with some segments being concave”. Gebien (1928) studied non-type material identified as *Gamaxus* and concluded that it should be a synonym of *Phymatestes*, stating that it agrees with *Phymatestes* in all essential characters. This synonymy was included in the catalog by Gebien (1941) and in a subsequent taxonomic study of *Phymatestes* (Ferrer and Moraguès 1998). However, several other catalogs and regional lists have treated *Gamaxus* as valid (Blackwelder 1945; Smith et al. 2015; Bouchard et al. 2021), although without any justification for reversing the synonymy.

During this study, the holotype of *Gamaxushauxwelli* Bates, 1868 (Fig. 29) was examined and the specimen clearly agrees with *Phymatestes* in several important characters. The large body size, parallel form, tuberculate elytra, and femora armed with spines are all consistent with species of *Phymatestes*. Furthermore, the shape of the femora and compact antennal segments are identical with *P.brevicornis* (Lacordaire, 1859) and thus *G.hauxwelli* Bates, 1868 is considered a new synonym of *P.brevicornis* (Lacordaire, 1859).

#### ﻿Genus Anaedus

Taxon classificationAnimaliaColeopteraTenebrionidae

Blanchard, 1842

8F8310BC-E759-514A-9D24-80853B48CCC7

Figs 30–41 , 42–45

Anaedus Blanchard, 1842: pl. 14. Type species: Anaeduspunctatissimus Blanchard, 1842 (Fig. 27).
= Aspisoma Duponchel & Chevrolat, 1841: 240. Type species: Aspisomafulvipenne Duponchel & Chevrolat, 1841. Synonymy by Lacordaire 1859: 396, junior homonym of Aspisoma Laporte, 1833 (Coleoptera: Lampyridae).
= Anaedes Agassiz, 1846: 20. Type species: Anaeduspunctatissimus Blanchard, 1842. Unjustified emendation, not in prevailing usage (Bouchard et al. 2021).
= Aspidosoma Agassiz, 1846: 36. Type species: Aspisomafulcipenne Duponchel & Chevrolat, 1841. Unjustified emendation, not in prevailing usage (Bouchard et al. 2021).
= Microanaedus Pic, 1923: 16. Type species: Microanaedusnotatus Pic, 1923. syn. nov. (Fig. 36).
= Pengalenganus Pic, 1917a: 10. Type species: Pengalenganusinaequalis Pic, 1917a. syn. nov.
= Pseudanaedus Gebien, 1921: 107. Type species: Pseudanaedusbiangulatus Gebien, 1921. syn. nov.(Fig. 37).
= Pseudolyprops Fairmaire, 1882: 236. Type species: Pseudolypropsdilaticollis Fairmaire, 1882. syn. nov. (Fig. 38).
= Spinolyprops Pic, 1917a: 12. Type species: Spinolypropsrufithorax Pic, 1917a. syn. nov. (Fig. 39).
= Spinadaenus Pic, 1921: 18. Type species: Spinadaenussingularis Pic, 1921. syn. nov. (Fig. 40).
= Sphingocorse Gebien, 1921: 110. Type species Sphingocorseangulicollis Gebien, 1921. syn. nov. (Fig. 41).
= Trichulodes Carter, 1914: 223. Type species: Trichulodespunctatus Carter, 1914. Synonymized with Pseudolyprops by Doyen et al. (1990: 231).

##### Diagnosis.

*Anaedus* may be generally differentiated from other Goniaderini by the following combination of characters: (1) eyes reniform, not completely divided; (2) pronotum transverse, always wider than long, never divided by narrow waist; (3) femora lacking teeth and spines; (4) tarsal formula 5-5-4; (5) elytra with basal lateral margin distinctly serrate; (6) elytral striae in most species, at least confused basally, usually confused throughout entire length.

*Anaedus* is most similar to *Aemymone*, *Lyprochelyda*, and *Ancylopoma*. From *Aemymone*, it can be distinguished by the setae on the lateral margin of the elytra placed on the lateral carina (in *Aemymone*, the setae on the lateral margin of the elytra are placed dorsad to the lateral carina). In most species of *Anaedus*, elytral punctures are nearly always confused (punctures always in linear striae in *Aemymone*). In *Anaedus*, the basal lateral margin of the elytron is distinctly serrate (Figs 42–44) whereas in *Aemymone*, the basal lateral margin of the elytron is smooth and never serrate (Fig. 45). *Lyprochelyda* possesses a wide, transverse pronotum and elytra with confused punctures similar to *Anaedus* but can be distinguished by the presence of a large tooth on the middle and hind femora. *Anyclopoma* possesses elytra with confused punctures like *Anaedus*, but the width of the base of the pronotum is shorter than the length of pronotum (see Johnston et al. 2022 for additional discussion). These three genera fall close to our expanded concept of *Anaedus* but seem to us recognizably distinct in the specimens at hand and are here retained as valid genera, though more data are desired to help clarify these relationships in the future.

**Figures 30–41.**
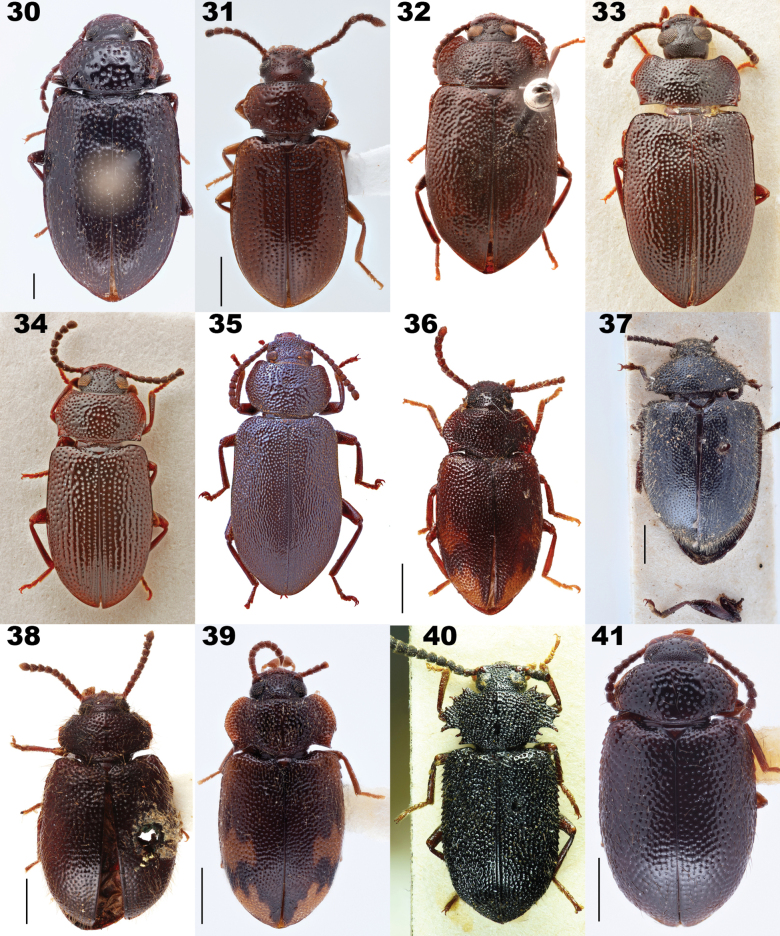
Dorsal habitus of species of *Anaedus* Blanchard, 1842 including species belonging to genera synonymized with *Anaedus* in this paper. **30***Anaeduspunctatissimus* Blanchard, 1842, type species of *Anaedus***31***A.brunneus* (Ziegler, 1844) **32***A.expansicollis* Gebien, 1913, paratype **33***A.explanatus* Pic, 1917 **34***A.leleupi* Ardoin, 1876, paratype **35***A.robusticollis* Pic, 1921 **36***A.notatus* (Pic, 1923), syntype, type species of *Microanaedus* Pic, 1923 **37***A.conradti* (Gebien, 1921), originally described in *Pseudanaedus* Gebien, 1921 **38***A.dilaticollis* (Fairmaire, 1882), holotype, type species of *Pseudolyprops* Fairmaire, 1882 **39***A.himalayicus* (Kaszab, 1965), originally described in *Spinolyprops* Pic, 1917 **40***A.serrimargo* (Gebien, 1914), senior subjective synonym of *Spinadaenussingularis* Pic, 1921, the type species of *Spinadaenus* Pic, 1921. **41***A.nepalicus* (Kaszab, 1975), originally described in *Sphingocorse* Gebien, 1921. Scale bars: 1 mm. Images lacking scale bars were produced by Otto Merkl and sizes of specimens were not recorded before he passed. Figs 36, 38 taken by Christophe Rivier (MNHN).

Distinguishing *Anaedus* and the newly synonymized genera has long been problematic. Characters initially used to distinguish these genera are here considered to be unreliable, especially when many species of this group were examined. Schawaller (2011) stated this problem, saying “the separation of the genera *Pseudolyprops* Fairmaire, 1882, *Sphingocorse* Gebien, 1921, and *Spinolyprops* Pic, 1917 within the tribe Lupropini[sic] is still in a preliminary state and not yet based on discriminating characters.” At that time, *Anaedus* was placed in Goniaderini and therefore Schawaller did not include it or other similar genera within Goniaderini in his discussion and analysis. With our newly updated tribal concepts, the delimitation of these genera required additional investigation.

We examined 66 species of our broadened concept of *Anaedus*, including the type species of all newly synonymized genera except *Pengaleganus*. We examined the characters purported to distinguish these groups and discuss them below under specific synonymies. The updated diagnosis above delimits our broad concept of *Anaedus* from other members of Goniaderini.

*Microanaedus* (Fig. 36), known from Sumatra and Gabon, was distinguished from *Anaedus* by its small size (roughly 5 mm) and the structure of the prothorax, which is described as transverse, laterally crenulate, regularly arched, with posterior corners prominent (Pic 1923). Both the size and the structure of the prothorax fall clearly within the range of *Anaedus*. Prominent hind angles are used as a character to distinguish other synonymized genera including *Spinolyprops*. Numerous examined *Anaedus* species also have this character, and thus it is not reliable for distinguishing genera in this complex. *Microanaedus* is placed as a synonym resulting in *Anaedusnotatus* (Pic, 1923), comb. nov. and *Anaedusbartolozzii* (Ferrer, 2002), comb. nov.

*Pengalenganus*, known from the Indomalayan region, was also distinguished from *Anaedus* by the structure of the pronotum, which was described as short, strongly incised anteriorly in the middle, with anterior angles prominent, very constricted posteriorly to the middle, laterally margined and flattened, and laterally posteriorly incised (Pic 1917a). Although we have not examined specimens attributed to this genus, the description of the pronotum falls within the diversity seen in *Anaedus*. Additionally, the synonym was first suggested by Kaszab in his unpublished annotations in his physical copy of the Gebien (1941) Catalog complemented with handwritten remarks “*Pengalenganus* Pic = *Anaedus*!” and his comment about the type species “9731 *inaequalis* Pic. Mel. Ent, 23, 1917, 10 Java = *Anaedus* 9759A.” He spent considerable time studying tenebrionid material deposited in the Muséum national d’Histoire Naturelle, Paris, and very likely saw Pic’s types. This synonymy results in *Anaedusinaequalis* (Pic, 1917a), comb. nov., *Anaedusangustatus* (Pic, 1921), comb. nov., and *Anaedustestaceicornis* (Pic, 1921), comb. nov.

*Pseudanaedus* (Fig. 37), with two species known from Cameroon, is characterized mainly by what Gebien considered to be a deep groove around the dorsal lobe of the eye. However, other species of *Anaedus* have grooves around the eyes to varying degrees. In South America, this feature seems more prevalent in species with a pronotum with spinose posterior angles. *Pseudanaedus* was also characterized by being hairy. This character state is also present in numerous *Anaedus* species, as well as newly synonymized genera (e.g., *Pseudolyprops* and *Spinolyprops*), and is not diagnostic. Therefore, *Pseudanaedus* Gebien, 1921 is placed as a synonym of *Anaedus* resulting in: *Anaedusbiangulatus* (Gebien, 1921), comb. nov. and *Anaedusconradti* (Gebien, 1921), comb. nov.

*Pseudolyprops* (Fig. 38), distributed in the Australasian and Indomalayan regions, is also distinguished by the shape of the pronotum (Fairmaire 1882; Wei and Ren 2020). Again, this is not a diagnostic character and *Pseudolyprops* is placed in synonymy with *Anaedus*, resulting in the following new combinations:

*Anaedusanaedoides* (Gebien, 1921), comb. nov.

*Anaedusalbipes* (Gebien, 1921), comb. nov.

*Anaedusamboinensis* (Kaszab, 1964), comb. nov.

*Anaedusamplicollis* (Fairmaire, 1896), comb. nov.

*Anaedusaustraliae* (Carter, 1930), comb. nov.

*Anaedusbeloni* (Fairmaire, 1888), comb. nov.

*Anaedusborneensis* (Pic, 1917b), comb. nov.

*Anaeduscarinicollis* (Gebien, 1921), comb. nov.

*Anaedusgabonicus* (Pic, 1917b), comb. nov.

*Anaedusjacobsoni* (Gebien, 1927), comb. nov.

*Anaeduslatus* (Pic, 1917b), comb. nov.

*Anaeduslongeplicatus* (Gebien, 1921), comb. nov.

*Anaedusmajor* (Pic, 1917b), comb. nov.

*Anaedusnigrita* (Gebien, 1927), comb. nov.

*Anaeduspinguis* (Gebien, 1927), comb. nov.

*Anaeduspunctatus* (Carter, 1914), comb. nov.

*Anaedusraffrayi* (Pic, 1917b), comb. nov.

*Anaedusrufus* (Pic, 1917b), comb. nov.

*Anaedussumatrensis* (Pic, 1917b), comb. nov.

*Anaedusterminatus* (Gebien, 1921), comb. nov.

*Anaedustestaceipes* (Pic, 1917b), comb. nov.

*Spinolyprops* (Fig. 39), known from Asia, was also considered to have a unique prothorax (Pic 1917a) and was characterized by the color patterning of the elytra. Again, the pronotum falls within the diversity seen in *Anaedus*. Patterned elytra are also seen in *Anaedus* species from multiple biogeographic realms as well as *Microanaedus* (Fig. 33) and thus is not a reliable character to distinguish genera. We place *Spinolyprops* as a synonym of *Anaedus*, resulting in the following new combinations, and necessitating two new replacement names.

*Anaedusrufithorax* (Pic, 1917a), comb. nov.

*Anaedusmaculipennis* nom. nov. for *Spinolypropsmaculatus* Kulzer, 1954: 21. Distribution: Sri Lanka. Secondary homonym of *Anaedusmaculatus* Champion, 1886: 25. Distribution: Nicaragua and Panama.

*Anaeduscribricollis* (Schawaller, 2012), comb. nov.

**Figures 42–45.**
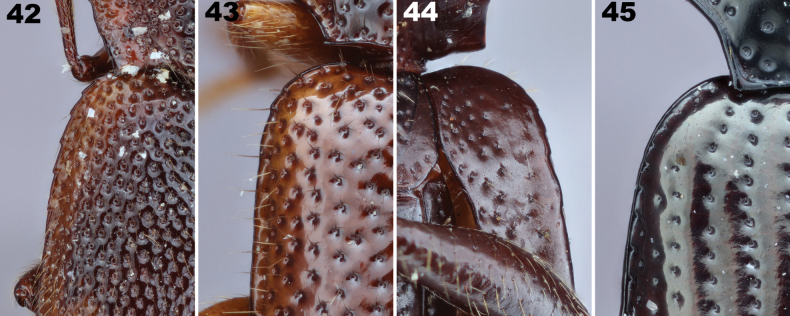
Elytral humerus of *Anaedus* and *Aemymone*. **42***Anaeduslateralis* (Pic, 1917), formerly in *Spinolyprops***43***Anaedusbrunneus* Ziegler, 1844 **44***Anaeduspunctatissimus* Blanchard, 1842 **45***Aemymone* sp.

*Anaedushimalayicus* (Kaszab, 1965), comb. nov.

*Anaeduslateralis* (Pic, 1917a), comb. nov.

*Anaedusottomerkli* nom. nov., for *Anaeduslateralis* Pic, 1923: 16. Distribution: Vietnam. Secondary homonym of *Anaeduslateralis* (Pic, 1917a: 12) [Spinolyprops]. Distribution: Myanmar, Thailand, Laos, Malaysia, and Indonesia.

*Anaeduspakistanicus* (Schawaller, 1996), comb. nov.

*Anaedusthailandicus* (Schawaller, 2012), comb. nov.

*Anaedustrautneri* (Schawaller, 1994), comb. nov.

*Spinadaenus* (Fig. 40), a monotypic genus known from Borneo, is unique with its extreme laterally spinose pronotum. Many species of *Anaedus* have a pronotum with undulate margins, and a few species, including *Anaedusserratus* Wei & Ren, 2020 have denticulate lateral margins. Although the pronotum of *Spinadaenus* is very spinose, we believe it represents a single extreme example of the range found within *Anaedus* and therefore the genus is placed as a synonym of *Anaedus* resulting in *Anaedusserrimargo* (Gebien, 1914), comb. nov. Note that the type species *Spinadaenussingularis* Pic, 1921 is considered a junior subjective synonym of *Lyprops* [sic] *serrimargo* Gebien, 1914 (Grimm and Schawaller 2021).

*Sphingocorse* (Fig. 41), known from Central Africa and Asia, was considered by Gebien to be very close to *Pseudanaedus*, differing in the shape of the penultimate hind tarsomere and absence of a deep groove around the top of the eye (Gebien 1921). In his key to African ‘Heterotarsinae’ (an old subfamily concept, which contained *Anaedus*, *Luprops*, and other genera considered to be similar), *Sphingocorse* and *Pseudanaedus* are distinguished from *Anaedus* by the shape of the pronotum. Again, the shape of the pronotum is not distinct and falls within the diversity of forms seen In *Anaedus*. We place *Sphingocorse* in synonymy with *Anaedus*, resulting in the following new combinations, and necessitating one new replacement name.

*Anaedusangulicollis* (Gebien, 1921), comb. nov.

*Anaedusnepalicus* (Kaszab, 1975), comb. nov.

*Anaedusmaculipennis* (Schawaller, 2011), comb. nov.

*Anaedusschawalleri* nom. nov. for *Anaedusnepalicus* Schawaller, 1994: 267. Distribution: Nepal. Secondary homonym of *Anaedusnepalicus* (Kaszab, 1975) [*Sphingocorse*]. Distribution: Nepal.

*Aspisoma* Duponchel & Chevrolat, 1841 (Coleoptera: Tenebrionidae) has a complicated taxonomic history, and although its synonymy with *Anaedus* was established by Lacordaire (1859) and has persisted to this day, we uncovered additional taxonomic issues concerning this name. The name ‘*Aspisoma*’ was published in Dejean’s (1834) second catalog as a genus belonging to Hétéromères: Ténébrionites but included no available species and thus is not available from that publication (Bousquet and Bouchard 2013). The name was validated by Duponchel and Chevrolat (1841) who, referring the name to Dejean, diagnosed the genus and included the type species *Aspisomafulvipenne* Duponchel & Chevrolat, 1841. However, the authors failed to realize that the name “*Aspisoma*” had already been published by Laporte (1833) for a genus of Lampyridae (Coleoptera), and thus *Aspisoma* Duponchel & Chevrolat, 1841 is a junior homonym of *Aspisoma* Laporte, 1833.

Several papers by Pic (1917b, 1917c, 1932, 1934) described 14 new species in the genus “*Aspisoma*” which have long been confused in catalogs and checklists. Gebien (1941) recognized these species as belonging to *Aspisoma* Duponchel & Chevrolat (Tenebrionidae) and therefore included them within the genus *Anaedus* following Lacordaire’s synonymy. However, Blackwelder (1945) listed all 14 Pic species in *Aspisoma* Laporte (Lampyridae) where they have continued to be listed (McDermott 1966). One of the species described by Pic (*Aspisomainangulata* Pic, 1934) was included as a member of *Anaedus* by Bousquet et al. (2018).

All four of Pic’s works indicate that the species were meant to be placed in Tenebrionidae. In each paper, the species are described between *Anaedus* and other genera which we here treat as synonyms (e.g., *Pseudolyprops*). Furthermore, Pic (1917b) compares one of his species to a species of *Anaedus*. We have not seen any of these types but from the descriptions and arrangement in his works we are confident that Pic meant to place these species in *Aspisoma* Duponchel & Chevrolat (Tenebrionidae), though it is not clear if he merely missed Lacordaire’s synonymy or truly intended to return the group to genus rank.

We recognize the following species as members of Tenebrionidae: Lagriinae which leaves no western hemisphere species described by Pic remaining in *Aspisoma* Laporte (Lampyridae). The combinations are restored to Gebien’s (1941) inclusion within *Anaedus* and results in the following nomenclatural acts:

*Anaedusboliviensis* (Pic, 1934: 36), comb. rest.

*Anaedusclaveri* (Pic, 1917c: 13), comb. rest.

*Anaedusdiversicollis* (Pic, 1917b: 22), comb. rest.

*Anaeduselongatus* (Pic, 1934: 36), comb. rest.

*Anaedusgrimmi* nom. nov. for *Aspisomaforticornis* Pic, 1917b: 23. Distribution: Brazil. Secondary homonym of *Anaedusforticornis* (Fairmaire, 1883: 35) [*Lyprops*]. Distribution: Indonesia. See Grimm and Schawaller 2021.

*Anaedusguyanensis* (Pic, 1917b: 22), comb. rest.

*Anaedusholtzi* (Pic, 1934: 36), comb. rest.

*Anaedusinangulatus* (Pic, 1934: 35), comb. rest.

*Anaedusinhumeralis* (Pic, 1917b: 24), comb. rest.

*Anaedusmendesensis* (Pic, 1917b: 23), comb. rest.

*Anaedusminutus* (Pic, 1917b: 24), comb. rest.

*Anaedusrufimembris* (Pic, 1932: 17), comb. rest.

*Anaedusrufipennis* (Pic, 1917b: 23), comb. rest.

*Anaedussubelongatus* (Pic, 1932: 17), comb. rest.

*Anaedusminutus* (Pic, 1917b: 24), comb. rest.

*Anaedusminimus* nom. nov. for *Anaedusminutus* Pic, 1938: 16. Distribution: Vietnam. Secondary homonym of *Anaedusminutus* (Pic, 1917b) [*Aspisoma*] Distribution: Brazil.

*Anaedusmerkli* nom. nov. for *Anaedusdiversicollis* Pic, 1938: 17. Distribution: Vietnam. Secondary homonym of *Anaedusdiversicollis* (Pic, 1917b: 22) [*Aspisoma*]. Distribution: Guyana.

#### ﻿Provisional key to the genera of Goniaderini

**Table:** 

1	Pronotum divided into two clear sections by a narrow “waist”, at least anterior section bearing a large elongate horn-like spine laterally [Afrotropical] (Fig. 24; also see Kaszab 1976)	***Spinolagriella***
–	Pronotum not divided as above	**2**
2	Eyes completely divided [Tropical America] (Fig. 26)	***Xanthicles***
–	Eyes not divided, typically reniform	**3**
3	At least some femora with teeth	**4**
–	All femora without teeth	**5**
4	Middle and hind femora with large tooth, pronotum more or less explanate laterally, dorsum reddish or patterned yellow and black, not tuberculate [Afrotropical] (Fig. 27)	***Lyprochelyda***
–	Profemur, sometimes other femora armed in males, surface metallic, tuberculate [tropical America] (Fig. 23)	***Phymatestes***
5	Anterior lateral angles of pronotum greatly extended forming posterior angled arcs with anterior margins with spinose extensions [tropical America] (Fig. 28; also see Johnston et al. 2022)	***Ancylopoma***
–	Anterior lateral angles of pronotum not greatly extended forming posterior angled arcs	**6**
6	Tarsal formula 5-5-4. Pronotum with lateral margin slightly concave before hind angles, hind angles variable	**7**
–	Tarsal formula 4-4-4. Pronotum with lateral angles evenly rounded without concave aspect near hind angles, hind angles obtuse, never spinose [tropical America] (see Kaszab 1973)	***Myrmecopeltoides***
7	Basal hind tarsomere distinctly longer than length of tarsomeres 2+4 or nearly as long as the other tarsomeres together	**8**
–	Basal hind tarsomere equal or subequal in size to terminal tarsomere	**9**
8	Lateral margin of elytra near humeral angle with setae placed in the marginal carina, rendering the margin interrupted and the outline variably serrate, if these serrations weak, punctures on elytral disc and apex always confused; elytral disc occasionally with punctures in distinct linear series (striate), but in those cases the lateral elytral margin distinctly serrate; widespread including Tropical America] (Figs 30–45)	***Anaedus***
–	Lateral margin of elytra with setae dorsad the lateral carina, not interrupting it; elytral disc and apex with punctures in linear series [tropical America] (Figs 21, 45)	***Aemymone***
9	Lateral margins of pronotum at most with one or two obtuse angles, slightly explanate in some species, not strongly explanate on anterior section, surface usually smooth but some species with small tubercles. Elytra striate to costate, some species with costae forming elongate or short tubercles, without large tubercles. Lateral aspect of elytra smooth, not strongly dentate [tropical America] (Fig. 22)	***Goniadera***
–	Lateral margins of pronotum and elytra strongly dentate, pronotum strongly explanate on anterior two-thirds. Both pronotum and elytra with numerous large tubercles [tropical America] (Fig. 25)	***Opatresthes***

##### Goniaderini not keyed

*Acropachia Mäklin, 1875 [Tropical America] One species, pronotum with lateral pits. We would have to see type to confirm tribe and key placement.

*Microgoniadera Pic, 1917a [Tropical America] One species, possibly another striate form of *Anaedus*.

#### ﻿Tribe Lupropini

Taxon classificationAnimaliaColeopteraTenebrionidae

Lesne, 1926

32B08D71-F681-5B3D-A4C9-12C39B03C30A

Figs 46–49 , 65

##### Type genus.

*Luprops* Hope, 1833.

##### Description.

Body length: 5.2–11.2 mm; stout to elongate, glabrous or setose. Most species are unicolored but some are bicolored (e.g., pronotum and elytra with different coloration).

***Head***: Eyes reniform, anteriorly notched by canthus, rarely completely divided. Antennae moderately long, usually reaching or slightly extending past base of pronotum; antennomeres obconical to moniliform.

***Thorax***: Pronotum shape variable, quadrate to cordate, usually narrower than width of elytra. Lateral margins complete. Procoxae clearly separated by prosternal process. Mesocoxal cavity laterally closed, at least partially, by mesepimeron. Elytra striate. Metathoracic wings well developed or absent. Legs slender, not fossorial, penultimate tarsomeres lobed or cupuliform.

***Abdomen***: Intersegmental membranes visible between sternites V–VII, abdominal hinging tenebrionoid. Defensive glands present (Fig. 65), gland reservoirs conical, lacking striations, reservoir openings wide. Ovipositor slender, with three to four clearly separated coxite lobes, terminal coxite digitate, gonostyli apical or subapical.

##### Diagnosis.

Lupropini can be distinguished from Goniaderini and Prateini by having the mesocoxal cavity open and abdominal defensive glands present.

In Lagriinae, this character combination is shared with Adeliini Kirby, 1828, Pycnocerini Lacordaire, 1859, and Lagriini. Lupropini can be distinguished from these tribes as follows:

Both Adeliini and Pycnocerini possess abdominal defensive glands, but their configuration is different from Lupropini. Adeliini defensive gland reservoirs open between sternites VIII and IX (Fig. 67) and Pycnocerini possesses just a single rectangular reservoir located medially between sternites VII and VIII (Fig. 66). In contrast, Lupropini has paired reservoirs that open between sternites VII and VIII (Fig. 65).

**Figures 46–49.**
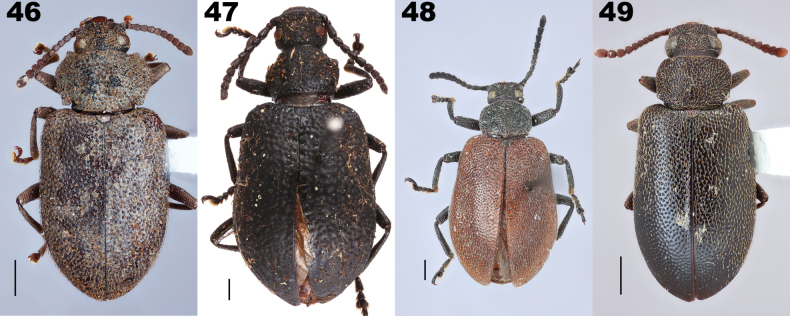
Dorsal habitus of representatives of Lupropini genera. **46***Coxelinus* sp. **47***Curtolypropslatipennis* Pic, 1917, syntype **48***Dichastopssubaeneus* Gerstaecker, 1871 **49***Lupropstristis* (Fabricius, 1801). Scale bars: 1 mm. Fig. 47 taken by Cristophe Rivier (MNHN).

Lagriini also possesses paired defensive gland reservoirs between sternites VII and VIII (Figs 62–64). This tribe is the most speciose in Lagriinae, and is currently divided into three subtribes: Lagriina Latreille, 1825, Statirina Blanchard, 1825, and Phobeliina Ardoin, 1961 (see below for justification of the inclusion of Phobeliina in Lagriini). The subtribes can be distinguished from Lupropini as follows. Lagriina is characterized by the terminal antennomere elongate in most species; prosternal process greatly reduced, resulting in the procoxae appearing to be nearly contiguous; pronotum lacks lateral carinae. Statirina is characterized by the terminal antennomere elongate in all species; prosternal process narrow or wide, clearly separating procoxae; pronotum has complete lateral carinae. Phobeliina is characterized by the terminal antennomere subequal to penultimate antennomere; prosternal process wide, clearly separating procoxae; pronotum lacks lateral carina. In contrast, Lupropini has terminal antennomere subequal to penultimate antennomere; prosternal process wide, clearly separating procoxae, pronotum with lateral carinae clearly developed, at least in anterior fourth.

##### Genera included.

*Coxelinus* Fairmaire, 1869, *Curtolyprops* Pic, 1917d, *Dichastops* Gerstaecker, 1871 and *Luprops* Hope, 1833.

#### ﻿Key to the genera of Lupropini

**Table:** 

1	Eyes completely divided by a broad epistomal canthus. [eastern and southern Africa] (monotypic *D.subaeneus* Gerstaecker, 1871 (Fig. 48; also see Schawaller 2011)	***Dichastops***
–	Eyes not divided (but sometimes narrowed) by a broad epistomal canthus	**2**
2	Pronotum with posterior margin notched subapically [Madagascar] (Fig. 46)	***Coxelinus***
–	Pronotum with posterior margin entire	**3**
3	Body relatively narrow, elongate; temples rounded, shorter than eye length in dorsal view; pronotum with lateral carinae thin, not ending with prominent process; pronotal and elytral surface regularly convex or flattened, not vermiculate [Afrotropical, Palearctic, Indo-Malaysian, Australian] (Fig. 49)	***Luprops***
–	Body very wide, elytra short; temples parallel-sided, rectangular posteriorly, longer than eye length in dorsal view; pronotum with lateral carinae thick, ending subanteriorly with prominent tooth-like process; pronotal and elytral surface coarsely uneven, vermiculate [Afrotropical] (Fig. 47)	***Curtolyprops***

### ﻿Miscellaneous notes on Lagriinae

#### ﻿Genus Capeluprops

Taxon classificationAnimaliaColeopteraTenebrionidae

Schawaller, 2011

2D13EC55-F5C3-5CD5-96F9-510AE386E613

Figs 3 , 50 , 52

Capeluprops Schawaller, 2011: 271. Type species: Capeluproprslaenoides Schawaller, 2011.

##### Note.

*Capeluprops* Schawaller, 2011 is provisionally moved from Lupropini to Laenini. *Capeluprops* contains six species of small, litter-inhabiting, flightless tenebrionids restricted to southern South Africa (Schawaller 2011). The genus was included in Lupropini without morphological discussion and the original description of the genus did not discuss the closure of the mesocoxal cavity nor presence or absence of defensive glands.

Paratypes and recently collected specimens of the type species were examined (Figs 4, 43, 45). The mesocoxal cavities of this species are open, as in Lupropini, but abdominal defensive glands are absent. Therefore, this genus is excluded from Lupropini. Five lagriine tribes share these two character states: Belopini, Chaerodini, Eschatoporini, Laenini, and Goniaderini. In *Capeluprops*, the presence of well-developed eyes, tenebrionoid abdominal hinging, and lack of highly modified adaptions for psammophily exclude it from the first three tribes. However, *Capeluprops* cannot be definitively placed in Laenini nor Goniaderini. As in all other known Laenini, *Capeluprops* lacks hind wings, and the elytra are fused. Although the eyes of *Capeluprops* (Fig. 45) are more developed than typical members of the tribe, the current definition of Laenini based on molecular and morphological data (Kanda 2016) includes species in South America with slightly reniform eyes (e.g., some species of *Chaetyllus* Pascoe, 1860 and *Grabulaxdarlingtoni*Kanda 2016). The ovipositor is very similar to those in Goniaderini, being very stout with long digitate gonocoxites. However, this character state is also present in a few Lupropini. Based on the absence of wings, and overall body form, we provisionally move *Capeluprops* to Laenini. Further data and a comprehensive review of Laenini are needed to confirm this placement.

#### ﻿Genus Plastica

Taxon classificationAnimaliaColeopteraTenebrionidae

Waterhouse, 1903

27A0197F-808F-57F8-A7C8-06D36CD5A996

Figs 51 , 53

Plastica Waterhouse, 1903: 563. Type species: Plasticapolita Waterhouse, 1903.

##### Note.

*Plastica* Waterhouse, 1903 is transferred from Apocryphini Lacordaire, 1859 (Tenebrioninae) to Laenini (Lagriinae). This genus contains a single species which occurs in high elevation arid regions around Lake Titicaca in Bolivia. Waterhouse (1903) placed *Plastica* in Apocryphini based on its apparent similarity to species in the genus *Apocrypha* Eschscholtz, 1831, but separated the two genera based on differences in the femora and tarsi. *Apocrypha*, as with all other members of Tenebrioninae, possess abdominal defensive glands that open between abdominal sternites VII and VIII.

**Figures 50–53.**
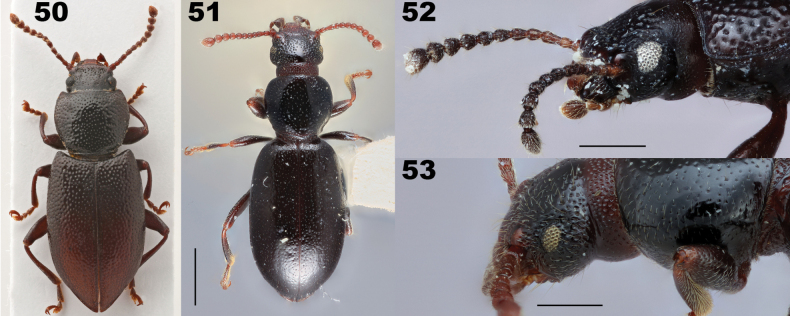
*Capeluprops* and *Plastica*, two genera transferred to Laenini. **50** Dorsal habitus of *Capelupropslaenoides* Schawaller, 2011, paratype **51** Dorsal habitus of *Plasticapolita* Waterhouse, 1903, specimen compared with holotype **52** lateral view of head of *C.laenoides*, non-type specimen **53** lateral view of head of *P.polita*, same specimen as Fig. 44. Scale bars: 1 mm (**51**), 0.5 mm (**52, 53**). Fig. 50 was produced by Otto Merkl and the size of the specimen was not recorded before he passed.

Examination of images of the holotype stored in the NHMUK provided by Dmitry Telnov and dissection of specimens matched with the holotype clearly place *Plastica* in Laenini. *Plasticapolita* does not possess abdominal defensive glands, excluding it from any lineages of Tenebrioninae. The following characters support its placement in Laenini: eyes small and round, not emarginate anteriorly (Fig. 53); mesocoxal cavity open; hind wings absent; elytral humeri rounded; abdomen with visible intersegmental membranes between abdominal sternites V–VII, lateral (tenebrionoid) hinging between these segments; abdomen lacking defensive glands.

#### ﻿Subtribe Phobeliina

Taxon classificationAnimaliaColeopteraTenebrionidae

Ardoin, 1961 stat. rev.

CE0889A3-AED0-56F8-A637-F7DC4102DCFC

Figs 56 , 57 , 60 , 61 , 64

##### Type genus.

*Phobelius* Blanchard, 1842.

##### Note.

*Phobelius* (Fig. 56) contains 13 Neotropical species. The genus was included in the group “Phobéliides” by Lacordaire (1859) within the tribe Hétérotarsides along with *Phymatestes*, *Anaedus*, and *Luprops*. Subsequently, Ardoin (1961) included Phobeliina as a subtribe of Adeliini and transferred all genera except *Phobelius* to other groups. Matthews (1998), in his comprehensive review of Adeliini, noted that *Phobelius* exhibits characters consistent with Lagriini and that the only difference between *Phobelius* and other members of the tribe was that *Phobelius* did not have the elongate terminal antennomere typically found in Lagriini. Matthews further concluded that *Phobelius* should be included in a third subtribe (separate from Lagriina and Statirina) in Lagriini. However, subsequent catalogus did not follow Matthews’ assessment, and *Phobelius* is currently included within Goniaderini with Phobeliina similarly synonymized under this tribe (Bouchard et al. 2005, 2011, 2021; Bousquet et al. 2018).

**Figures 54–57.**
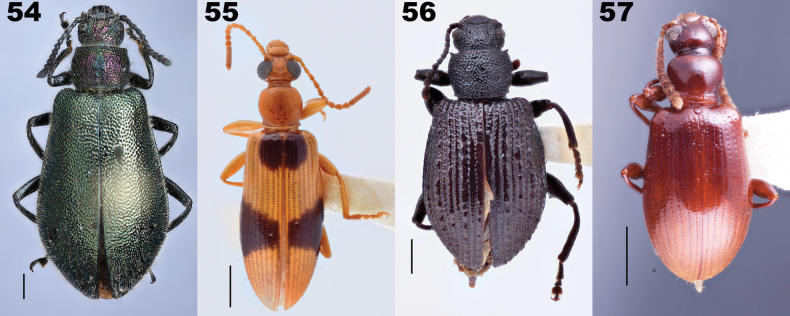
Dorsal habitus of representatives of Lagriini subtribes. **54***Lagriavillosa* (Fabricius, 1781), Lagriina**55***Statirapulchella* Mäklin, 1864, Statirina**56***Phobeliuslucifugus* Fairmaire, 1889, Phobeliina**57***Rhosacesclavipes* Champion, 1889, Phobeliina. Scale bars: 1 mm.

In molecular phylogenetic studies that included *Phobelius* (Kanda et al. 2015; Aalbu et al. 2017), the genus was recovered in a clade with Lagriini (Fig. 1), supporting Matthews’ conclusion. Lagriini (Figs 54–57) can be distinguished from other Lagriinae by the following characters: presence of abdominal defensive glands that open between abdominal sternites VII and VIII; pronotum with lateral margins absent or weakly impressed; antennae usually with terminal antennomeres elongate. Although *Phobelius* does not have elongate terminal antennomeres, they do have abdominal defensive glands and lack lateral pronotal margins.

**Figures 58–61.**
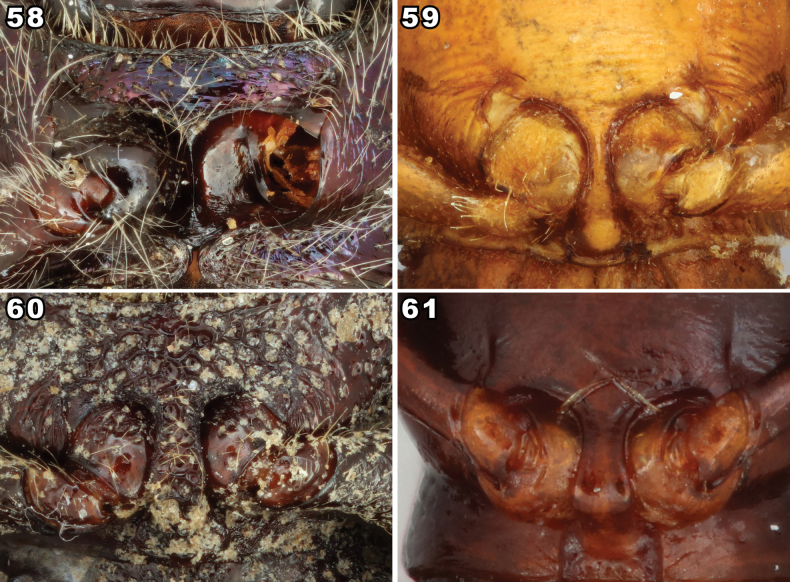
Prosterna of Lagriini. **58***Lagriavillosa* (Fabricius, 1781) **59***Statiragagatina* Melsheimer, 1845 **60***Phobelius* sp. **61***Rhosaces* sp.

Before this study, two subtribes of Lagriini were recognized, Lagriina and Statirina. The two subtribes can be distinguished based on differences in the prothorax. In Lagriina, the lobes of the hypomera meet behind the procoxae (Fig. 58) and, in Statirina, the lobes of the hypomera do not meet and are separated by the prosternum (Fig. 59). The prosternal process in Lagriina is thin and recessed between strongly projecting procoxae, sometimes resulting in the procoxal cavities appearing to be contiguous. In Statirina, the prosternal process forms a complete strip of cuticle, approximately ¼ the width of the procoxa, and clearly separates the coxae throughout their entire length. In addition to prothoracic characters, Lagriina tend to be broader bodied while Statirina tend to be more slender. The elongation of the terminal antennomere tends to be much more pronounced in Statirina, and in some Lagriina the terminal antennomere is nearly the same length as the penultimate antennomere.

**Figures 62–67.**
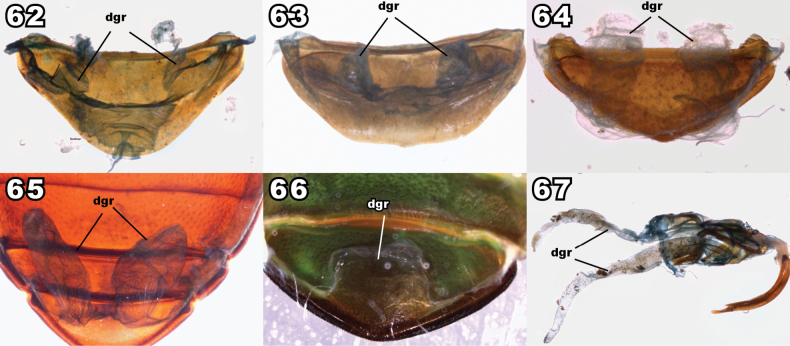
Defensive gland reservoirs of Lagriinae. **62***Lagriavillosa* (Fabricius, 1781), Lagriini: Lagriina**63***Statira* sp., Lagriini: Statirina**64***Phobelius* sp., Lagriini: Phobeliina**65***Luprops* sp., Lupropini**66***Aediotorix* sp., Pycnocerini**67***Cardiothorax* sp., Adeliini. Abbreviation: dgr = defensive gland reservoir.

As Matthews (1998) noted, *Phobelius* does not neatly fit within either of the two subtribes. Its prothorax (Fig. 60) resembles Statirina; the lobes of the hypomera do not meet posterior to the coxae and the prosternal process is wide and not recessed as in Lagriina. The stout body (Fig. 56) is more like body forms seen in Lagriina. The terminal antennomere is also not particularly elongate in either males or females, at most only 1.5 times longer than the preceding one. The shape of the abdominal defensive gland reservoirs differs from both Lagriina and Statirina as well. In *Phobelius*, the gland reservoirs are large and conical, with wide openings (Fig. 64). In Lagriina and Statirina, the gland reservoirs are small, sometimes inconspicuous, and are widely separated (Figs 62, 63).

We reinstate Phobeliina Ardoin, 1961 as a valid subtribe of Lagriini based upon the previous molecular phylogenetic analyses and morphological discussion presented above. We propose the following diagnosis of this lineage of Lagriini: body form stout; antennomeres stout, terminal antennomere not distinctly elongated in either sex; pronotum lacking lateral margin; procoxae separated by distinct prosternal process; hypomera extending mesally behind procoxae and both joined to prosternal process, not meeting each other; mesocoxae open; paired defensive glands present between abdominal sternites VII and VIII, glands large, conical.

Based upon our updated recognition of Phobeliina, we also tentatively include within it the genus *Rhosaces* Champion, 1889 (Figs 57, 61). This monotypic genus was erected for *Rhosacesclavipes* Champion, 1889 and placed within Statirina where it has been treated ever since (Blackwelder 1945; Bousquet et al. 2018; Bouchard et al. 2021), although Champion (1889) pointed out the strong differences in antennae (lacking an elongate terminal antennomere), a short epistoma, and a broadly rounded intercoxal process of the abdomen. All of the characters mentioned by Champion (1889) are shared with *Phobelius*, and the defensive glands, mesocoxal openings, and prothoracic characters similarly seem to unite these two genera. It is clear that *Rhosaces* does not fit within our concept of Lagriina, and it does adhere to our diagnosis of Phobeliina, and we look forward to future phylogenetic investigations that can more rigorously test the monophyly of this assemblage.

#### ﻿Genus Paralorelopsis

Taxon classificationAnimaliaColeopteraTenebrionidae

Marcuzzi, 1994

8B59B590-F640-5301-93EC-B49427841A3E

Paralorelopsis Marcuzzi, 1994: 117. Type species: Paralorelopsisbordoni Marcuzzi, 1994.

##### Note.

Marcuzzi (1994), in his very limited description based on a single example, described *Paralorelopsis* as agreeing with Champion’s description of *Lorelopsis* except for a single difference being the lack of a lamina on the subapical tarsomere. His new species, *P.bordoni*, is also much larger in size than species of either *Lorelopsis* or *Prateus*. In both the tarsi and size, it agrees more with some American genera belonging to Belopini. We were unable to examine specimens of this genus and therefore place it as incertae sedis in Lagriinae for now.

### Taxa excluded from Lagriinae

#### ﻿Genus Pseudesarcus

Taxon classificationAnimaliaColeopteraTenebrionidae

Champion, 1913

F883C19A-B9C1-58B9-B093-0688C77F0834

Figs 61 , 62–66

Pseudesarcus Champion, 1913: 115. Type species: Pseudesarcusvillosus Champion, 1913.

##### Note.

**Figures 68–73.**
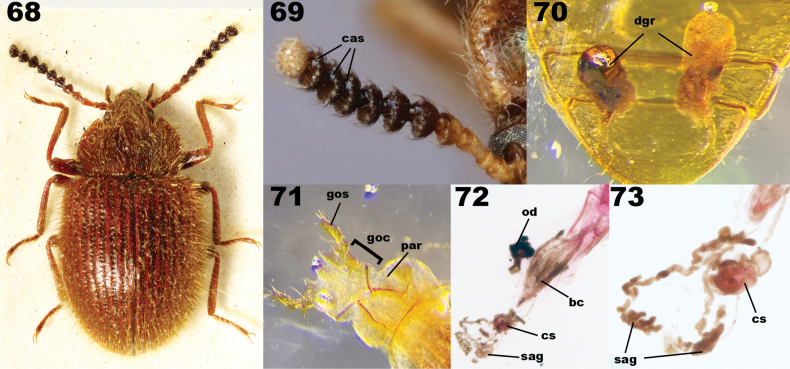
Dorsal habitus and structures of *Pseudesarcus* Champion, 1913. **68** Dorsal habitus of *Pseudesarcusvillosus* Champion, 1913, holotype **69** antennae of *Pseudesarcus* sp. **70** defensive gland reservoirs of *P.villosus***71** ovipositor of *P.villosus***72** female internal reproductive tract of *Pseudesarcus* sp. **73** details of spermatheca/spermathecal accessory gland complex of *Pseudesarcus* sp. Abbreviations: cas = compound antennal sensoria, dgr = defensive gland reservoir, par = paraprocts, goc = gonocoxites, gos = gonostyli, od = oviduct, bc = bursa copulatrix, cs = capsular spermatheca, sag = spermathecal accessory gland. Fig. 68 taken by Keita Matsumoto (NHMUK).

*Pseudesarcus* is placed incertae sedis within Diaperinae. *Pseudesarcus* was described in the family Mycetophagidae and transferred to Lagriinae incertae sedis by Lawrence and Newton (1995: 886) (Bousquet et al. 2018). *Pseudesarcusvillosus* was described from two Panamanian specimens, one of which was photographed by Keita Matsumodo (Fig. 68) and the other examined for us by Maxwell Barclay (both from NHMUK). A third specimen from Costa Rica (Figs 70–73) was identified as this genus based upon the images and description of the types and was dissected to examine internal structures. We also identified a seemingly undescribed species from Ecuador that possessed internal and external characters used to diagnose *Pseudesarcus* (Fig. 69). *Pseudesarcus* is clearly a member of Tenebrionidae and part of the ‘tenebrionoid-branch’ (sensu Doyen and Tschinkel 1982; see Matthews and Bouchard 2008).

*Pseudesarcus* can be characterized by: stellate antennal sensoria present on antennomeres 5–11 (Fig. 69); labrum concealed beneath epistoma with symmetrical epistomal tormae; lacinia lacking uncus; procoxal cavities closed internally and externally; mesocoxae closed laterally by mesoventrite and metaventrite; paired defensive glands present, lacking common volume, not pleated (Fig. 70); female genital tract with secondary bursa copulatrix, spermatheca forming annulated sclerotized capsule at end of spermathecal gland; ovipositor reduced (Figs 71–73).

Based on the above observations, *Pseudesarcus* is clearly not a lagriine (possesses stellate sensoria, lacks internal ridge of sternite VII) and seems to fall within the circumscription of Diaperinae (see Doyen and Tschinkel 1982; Matthews and Bouchard 2008; Johnston et al. 2020), but lacks any clear relationships with the established tribes (see Johnston et al. 2020). We place it as incertae sedis within Diaperinae until such time as its constituent tribes are better understood.

#### ﻿Genus Falsotithassa

Taxon classificationAnimaliaColeopteraTenebrionidae

Pic, 1934

D35F7A68-EA0D-58DD-A07C-BF1C0D8AB2EA

Figs 74–78

Falsotithassa Pic, 1934: 18. Type species: Falsotithassasumatrana Pic, 1934.

##### Note.

*Falsotithassa* Pic, 1934 is transferred from Lupropini (Lagriinae) to Leiochrinini Lewis, 1894 (Diaperinae). *Falsotithassa* contains ten species of small Tenebrionidae distributed across the Indo-Malayan biogeographic region. In the original description of this genus, Pic noted its similarity to *Tithassa*, which in this present paper is classified in Prateini. Based on the ordering of the descriptions in Pic’s (1934) manuscript, and the placement of the descriptions of *Falsotithassa* between species of *Anaedus* (Goniaderini) and *Tithassa*, it can be inferred that Pic considered this genus to be closely related to these taxa, and therefore included in Lagriinae. Schawaller (2000) revised *Falsotithassa*, providing a detailed diagnosis for this genus, and synonymized *Derispiolina* Kaszab, 1979 which was originally described as a tentative member of the tribe Leiochrinini (Kaszab 1979). In that study, Schawaller suggested that *Falsotithassa* belonged in Diaperini (Diaperinae), but in a later paper (Schawaller 2007c) reinterpreted the same characters studied in his initial 2000 revision as supporting the placement of *Falsotithassa* in Lupropini. This placement is adopted in the recent generic catalog of Tenebrionidae (Bouchard et al. 2021).

Examination of character states not discussed by Schawaller (2000, 2007c) and reinterpretation of female reproductive structures described in these papers support the exclusion of *Falsotithassa* from Lagriinae and supports its inclusion in Leiochrinini. The most evident character for excluding *Falsotithassa* from Lagriinae is the presence of complex sensoria on antennomeres 4–11 (Fig. 75); all Lagriinae have only simple antennal sensoria. Additionally, the abdominal defensive gland reservoirs have a lateral commissure joining the base of the left and right reservoir (Fig. 76). This arrangement is only known to occur in Leiochrinini and Nilionini (Doyen et al. 1990; Matthews and Bouchard 2008). The main characters used by Schawaller (2000, 2007c) in placing *Falsotithassa* in Diaperini was the presence of a capsular spermatheca (“check valve”), a character that is only known to occur in Diaperini and Nilionini (Tschinkel and Doyen 1980; Aloquio and Lopes-Andrade 2016). New dissections of specimens identified as *Falsotithassasumatrana* by Schawaller, and matched with the holotype, show a large thin-walled balloon-like spermathecae (Figs 77, 78). These are not the same as the capsular spermathecae present in Diaperini and Nilionini but are very similar to spermathecae illustrated for other species of Leiochrinini (Doyen et al. 1990; Matthews and Bouchard 2008).

**Figures 74–78.**
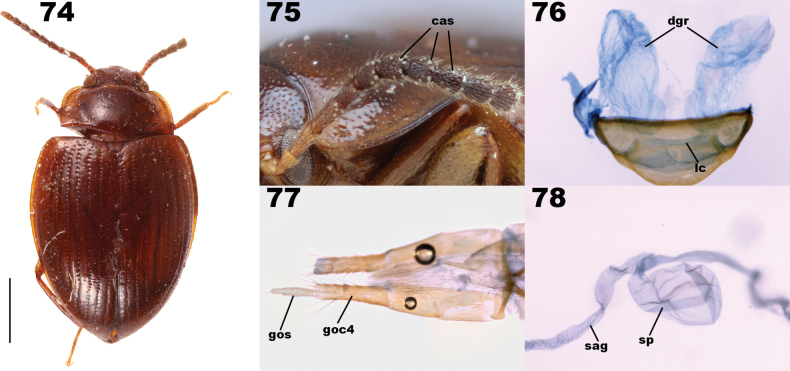
Dorsal habitus and structures of *Falsotithassasumatrana* Pic, 1934. **74** Dorsal habitus of holotype **75** antennae of non-type specimen **76** defensive gland reservoirs **77** ovipositor **78** portion of spermatheca. Abbreviations: cas = compound antennal sensoria, dgr = defensive gland reservoir, lc = lateral commissure, goc4 = fourth gonocoxite, gos = gonostylus, sp = spermatheca, sag = spermathecal accessory gland. Fig. 74 taken by Cristophe Rivier (MNHN). Scale bar: 1 mm.

We transfer *Falsotithassa* to Leiochrinini based upon the three characters discussed above: (1) antennae with complex sensoria on antennomeres 4–11; (2) abdominal defensive gland reservoirs joined by lateral commissure; (3) female internal reproductive tract with large thin-walled spermathecae. Further support of this conclusion is provided by characters mentioned by Schawaller (2000), including internally open procoxal cavities and the female reproductive tract lacking a bursa copulatrix. However, *Falsotithassa* departs from the coccinellid-like appearance of all other current members of Leiochrinini and indeed is externally similar to members of Scaphidemini Reitter, 1922. The latter is presently defined by a strongly sclerotized T-shaped spermatheca and the defensive glands lacking a commissure (Doyen et al. 1990; Matthews and Bouchard 2008) which preclude the placement of *Falsotithassa* therein. We hypothesize that the tribes Leiochrinini and Scaphidemini are likely closely related and should be reevaluated with respect to each other in future studies.

#### ﻿Genus Mimocellus

Taxon classificationAnimaliaColeopteraTenebrionidae

Wasmann, 1904

43B4F7AC-6625-57C8-AF60-3CE7422FEE80

Figs 79–81

Mimocellus Wasmann, 1904: 11. Type species: Mimocellustrechoides Wasmann, 1904: 12.

##### Note.

*Mimocellus* is placed incertae sedis in Tenebrionidae belonging in either Diaperinae or Stenochiinae. *Mimocellus* contains seven sub-Saharan African species of Tenebrionidae, including several species that are associated with termite nests. In the original description, Wasmann (1904), on the advice of E. von Oertzen, placed the genus near *Luprops* within the composite group ‘Heterotarsini’ based on unspecified similarities in head morphology. More recent treatments of the genus and taxonomic catalogs place *Mimocellus* in Lupropini (Schawaller 2005; Robiche et al. 2002; Bouchard et al. 2021).

**Figures 79–81.**
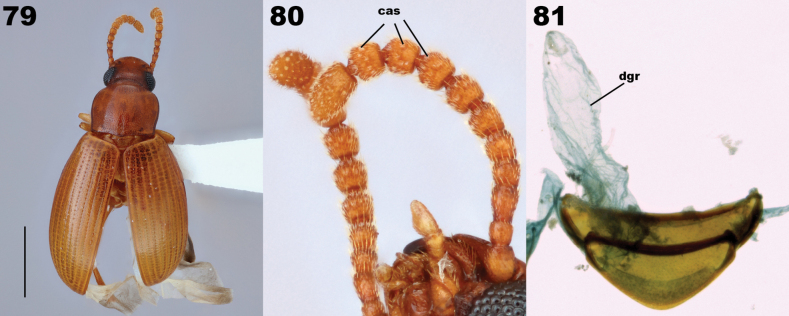
Dorsal habitus and structures of *Mimocellus* Wasmann, 1904. **79** Dorsal habitus of *Mimocellustrechoides* Wasmann, 1904 **80** antennae of *M.trechoides***81** defensive gland reservoir of *M.trechoides*, one reservoir was damaged during dissection. Abbreviations: dgr = defensive gland reservoir. Scale bar: 1 mm.

As with *Falsotithassa*, examination of antennae clearly excludes *Mimocellus* from Lagriinae. *Mimocellus* has distinct patches of complex antennal sensoria (Fig. 80). Additionally, the defensive gland reservoirs differ from those found in Lupropini. While lupropine gland reservoirs tend to be stout with wide openings (Fig. 65), *Mimocellus* possesses elongate gland reservoirs (Fig. 81) similar to the type found in Diaperini (Diaperinae) and Cnodalonini (Stenochiini) (Tschinkel and Doyen 1980). Due to limited availability of specimens, we were unable to examine female internal morphology. For this reason, we currently place this genus as incertae sedis within Tenebrionidae until its placement within the tenebrionoid-branch subfamilies can be elucidated.

#### ﻿Tribe Archaeolupropini

Taxon classificationAnimaliaColeopteraTenebrionidae

Nabozhenko, Perkovsky & Nazarenko, 2023

10E3DC69-541C-508A-9A3D-BDE6546936F7

##### Type genus.

*Archaeoluprops* Nabozhenko, Perkovsky & Nazarenko, 2023.

##### Note.

The tribe Archaeolupropini Nabozhenko, Perkovsky & Nazarenko, 2023 is transferred from Lagriinae to Tetratomidae: Tetratominae Billberg, 1820. This tribe was recently described for a single beetle preserved in Eocene amber (Nabozhenko et al. 2023). This beetle was compared to several tribes of Lagriinae where it was found to not belong to any of them, and therefore was placed into a new tribe in the subfamily. Examination of the descriptions and excellent photographs in that paper demonstrate that this taxon clearly belongs within the family Tetratomidae.

*Archaeolupropsgroehni* Nabozhenko, Perkovsky & Nazarenko, 2023 possesses the following characters consistent with Tetratomidae: the basal two ventrites connate with 3–5 articulated; antennal insertions visible from above; elongate and linear terminal maxillary palpomeres; vertical lateral aspect of the abdominal ventrites which fit beneath the elytra; paired depressions near the posterior pronotal margin; hind coxae elongate, not bounded laterally by the sides of the first abdominal ventrite. The images do not clearly show the procoxal closure, but it appears they could be open externally. The lack of elytral striae, shape of the scutellar shield, and general facies indicate that this species belongs in the nominate subfamily Tetratominae, though the available specimen does not allow for examination of antennal clubs or male genitalia which are the primary features currently used to separate tetratomid subfamilies (Nikitsky 1998). Based upon the preponderance of evidence, we hereby transfer Archaeolupropini to Tetratomidae: Tetratominae and leave it there as a valid tribe in that subfamily pending further revision.

#### ﻿Key to the extant tribes and subtribes of Lagriinae (Lagriinae also includes the extinct tribe Gonialaenini; see Nabozhenko et al. 2019)

**Table:** 

1	Mesocoxal cavities closed (i.e., meso- and metaventrites fully enclosing mesocoxal cavity)	**2**
–	Mesocoxal cavities open (i.e., laterally at least partially closed by mesepimeron)	**3**
2	Pronotum strongly flanged, covering head; abdominal membranes not exposed [Afrotropical, Palearctic, Indo-Malaysian]	**Cossyphini**
–	Head always visible from above, pronotum without flanges covering the head; abdomen with exposed membranes between sternites V–VII [worldwide]	**Prateini**
3	All species with eyes, eyes typically reniform	**5**
–	Few or all species without eyes, if eyes present, eyes rounded, globose; lacking sternal defensive glands	**4**
4	All species completely lacking eyes [Nearctic, associated with subterranean streams]	**Eschatoporini**
–	Almost all species with rounded eyes, which may be reduced in size and sometimes absent [Palearctic, Neotropical, Afrotropical, Indo-Malayan]	**Laenini**
5	Penultimate protarsomeres simple, not ventrally prolonged into lobes	**6**
–	Penultimate protarsomeres prolonged ventrally into lobes	**7**
6	Intersegmental membrane not visible between abdominal sternites; defensive glands absent [Nearctic, Palearctic, Neotropical and Australia]	**Belopini**
–	Intersegmental membrane clearly visible between abdominal sternites; single defensive gland between sternites VII and VIII [Afrotropical and Indo-Malayan]	**Pycnocerini**
7	Body globose; legs fossorial [Australasian, Australia, New Zealand, on beaches]	**Chaerodini**
–	Body not globose; legs not fossorial	**8**
8	Prosternal process very narrow, procoxae nearly contiguous [worldwide except Nearctic]	**Lagriini: Lagriina**
–	Prosternal process always visible between procoxae	**9**
9	Terminal antennomere very elongate especially in males [worldwide except Europe]	**Lagriini: Statirina**
–	Terminal antennomere normal length in both sexes	**10**
10	Pronotum lacking carina separating pronotum from epipleura [Neotropical]	**Lagriini: Phobeliina**
–	Pronotum always with carina separating pronotum from epipleura	**11**
11	Abdominal defensive glands present	**12**
–	Abdominal defensive glands absent [worldwide]	**Goniaderini**
12	Abdominal defensive glands paired between sternites VIII and IX [Neotropical and Australasian]	**Adeliini**
–	Abdominal defensive glands paired between sternites VII and VIII [Afrotropical, Palearctic, Indo-Malayan, Australasian, Oceanic]	**Lupropini**

A list of the proposed changes from the current positions of pertinent genera within Lagriinae are summarized in Table 1.

**Table 1.** Genera treated or figured in this study with previous classification, proposed taxonomic changes, and figure number.

Genus	Previous classification	Taxonomic changes	Figure(s)
*Acropachia* Mäklin, 1875	Goniaderini	–	–
*Aemymone* Bates, 1868	Goniaderini (subgenus of *Goniadera*)	Goniaderini (valid genus)	21, 45
*Aediotorix* Bates, 1868	Pycnocerini	–	66
*Anaedus* Blanchard, 1842	Goniaderini	–	30–44
*Ancylopoma* Pascoe, 1871	Goniaderini	–	28
*Antennoluprops* Schawaller, 2007	Lupropini	Prateini	–
*Archaeoluprops*Tetratominae Nabozhenko, Perkovsky & Nazarenko, 2023	Archaeolupropini (Tenebrionidae: Lagriinae)	Archaeolupropini (Tetratomidae: Tetratominae)	–
*Ardoiniellus* Schawaller, 2013	Lupropini	Prateini	–
*Bolitrium* Gebien, 1914	Lupropini	Prateini	6
*Capeluprops* Schawaller, 2011	Lupropini	Laenini	4, 50, 52
*Cardiothorax* Motschulsky, 1860	Adeliini	–	67
*Coxelinus* Fairmaire, 1869	Lupropini	–	46
*Curtolyprops* Pic, 1917	Lupropini	–	47
*Dichastops* Gerstaecker, 1871	Lupropini	–	48
*Enicmosoma* Gebien, 1922	Lupropini	Prateini	7
*Falsotithassa* Pic, 1934	Lupropini	Leiochrinini (Diaperinae)	74–78
*Gamaxus* Bates, 1868	Goniaderini	Goniaderini (synonym of *Phymatestes*)	29
*Goniadera* Perty, 1830	Goniaderini	–	22
*Indenicmosoma* Ardoin, 1964	Lupropini	Prateini	8
*Iscanus* Fauvel, 1904	Lupropini	Prateini	9
*Kuschelus* Kaszab, 1982	Lupropini	Prateini	–
*Lagria* Fabricius, 1775	Lagriini: Lagriina	–	54, 58, 62
*Lorelopsis* Champion, 1896	Lupropini (synonym of *Lorelus*)	Prateini (valid genus)	17–20
*Lorelus* Sharp, 1876	Lupropini	Prateini (synonym of *Prateus*)	–
*Luprops* Hope, 1833	Lupropini	–	49, 65
*Lyprochelyda* Fairmaire, 1899	Goniaderini	–	27
*Mesotretis* Bates, 1872	Lupropini	Prateini	10
*Microanaedus* Pic, 1923	Goniaderini	Goniaderini (synonym of *Anaedus*)	36
*Microcalcar* Pic, 1925	Lupropini	Prateini	11
*Microgoniadera* Pic, 1917a	Goniaderini	–	–
*Microlyprops* Kaszab, 1939	Goniaderini	Prateini (synonym of *Micropedinus*)	–
*Micropedinus* Lewis, 1894	Lupropini	Prateini	5, 12
*Mimocellus* Wasmann, 1904	Lupropini	Tenebrionidae incertae sedis	79–81
*Opatresthes* Gebien, 1928	Goniaderini (subgenus of *Goniadera*)	Goniaderini (valid genus)	25
*Paralorelopsis* Marcuzzi, 1994	Lupropini	Lagriinae incertae sedis	–
*Paratenetus* Spinola, 1845	Goniaderini	Prateini	13
*Pengalenganus* Pic, 1917	Goniaderini	Goniaderini (synonym of *Anaedus*)	–
*Phobelius* Blanchard, 1842	Goniaderini	Lagriini: Phobeliina	56, 60, 64
*Phymatestes* Pascoe, 1866	Goniaderini	–	3, 23, 29
*Plastica* C.O. Waterhouse, 1903	Apocryphini	Laenini	51, 53
*Prateus* LeConte, 1862	Goniaderini	Prateini	2, 15, 16
*Pseudanaedus* Gebien, 1921	Goniaderini	Goniaderini (synonym of *Anaedus*)	37
*Pseudesarcus* Champion, 1913	Lagriinae incertae sedis	Diaperinae incertae sedis	68–73
*Pseudolyprops* Fairmaire, 1882	Goniaderini	Goniaderini (synonym of *Anaedus*)	38
*Rhosaces Champion*, *1889*	Lagriini: Statirina	Lagriini: Phobeliina	57, 61
*Sphingocorse* Gebien, 1921	Lupropini	Goniaderini (synonym of *Anaedus*)	41
*Spinadaenus* Pic, 1921	Goniaderini	Goniaderini (synonym of *Anaedus*)	40
*Spinolagriella* Pic, 1955	Lupropini	Goniaderini	24
*Spinolyprops* Pic, 1917	Lupropini	Goniaderini (synonym of *Anaedus*)	39, 42
*Statira* Lepeletier & Audinet-Serville, 1828	Lagriini: Statirina	Lagriini: Statirina	55, 59, 63
*Terametus* Motschulsky, 1869	Lupropini	Prateini	–
*Tithassa* Pascoe, 1860	Goniaderini	Prateini	14
*Xanthicles* Champion, 1886	Goniaderini	–	26

## Supplementary Material

XML Treatment for Prateini

XML Treatment for Prateus

XML Treatment for Micropedinus

XML Treatment for Lorelopsis

XML Treatment for Goniaderini

XML Treatment for Aemymone

XML Treatment for Opatresthes

XML Treatment for Phymatestes

XML Treatment for Anaedus

XML Treatment for Lupropini

XML Treatment for Capeluprops

XML Treatment for Plastica

XML Treatment for Phobeliina

XML Treatment for Paralorelopsis

XML Treatment for Pseudesarcus

XML Treatment for Falsotithassa

XML Treatment for Mimocellus

XML Treatment for Archaeolupropini

## ﻿Additional information

### Author ORCIDs

Kojun Kanda https://orcid.org/0000-0001-5561-8471

Ottó Merkl https://orcid.org/0000-0003-3301-273X

Michael A. Ivie https://orcid.org/0000-0003-0996-2946

M. Andrew Johnston https://orcid.org/0000-0002-0166-6985

### Data availability

All of the data that support the findings of this study are available in the main text.
